# The Role of Cognitive Functioning in the ICF Framework: A Systematic Review of Its Influence on Activities and Participation and Environmental Factors in People with Cerebral Palsy

**DOI:** 10.3390/jcm14186393

**Published:** 2025-09-10

**Authors:** María Carracedo-Martín, Paula Moral-Salicrú, Montse Blasco, Marina Fernández-Andújar, Roser Pueyo, Júlia Ballester-Plané

**Affiliations:** 1 Faculty of Health and Life Sciences, Universitat Abat Oliba CEU, CEU Universities, Calle Bellesguard 30, 08022 Barcelona, Spain; mcarracedom1@uao.es (M.C.-M.); mblascos@uao.es (M.B.); mfernandezan@uao.es (M.F.-A.); jballesterp897@uao.es (J.B.-P.); 2 Department of Clinical Psychology and Psychobiology, Universitat de Barcelona, Passeig de la Vall d’Hebron 171, 08035 Barcelona, Spain; pmoralsa@ub.edu; 3 Institut de Neurociències, Universitat de Barcelona, Passeig de la Vall d’Hebron, 171, 08035 Barcelona, Spain; 4 Institut de Recerca Sant Joan de Déu, Carrer de Santa Rosa, 39-57, 08950 Esplugues de Llobregat, Spain

**Keywords:** cerebral palsy, cognition, activities and participation, environmental factors, international classification of functioning, systematic review

## Abstract

**Background/Objectives**: Cerebral palsy (CP) is the most common cause of motor disability in childhood and is frequently associated with cognitive impairments that limit autonomy and participation. While motor function is a known predictor of functional outcomes, the specific contribution of cognitive domains within the International Classification of Functioning, Disability and Health (ICF) framework remains unexplored. This systematic review examines the relationship between cognitive domains and the ICF components of Activities and Participation, and Environmental Factors in people with CP. **Methods**: Following PRISMA guidelines, a systematic search was conducted across six databases (PubMed, PsycINFO, CENTRAL, CINAHL, ERIC, and WOS) for studies published between 2002 and 2025. Eligible studies included participants with CP (*n* = 3056) and analyzed associations between cognitive functions and ICF domains using standardized tools and statistical methods. Risk of bias was evaluated using the Oxford Centre for Evidence-Based Medicine criteria. **Results**: Forty-four studies met inclusion criteria, involving mostly children and adolescents with spastic CP and mild to moderate motor impairment. General intellectual functioning, language, and visual perception were the most studied domains, showing consistent associations with ICF chapters such as Learning and applying knowledge, Communication, and Mobility. Although fewer studies examined Environmental Factors, relevant associations emerged with support systems, attitudes, and services. Heterogeneity in assessment methods and participant profiles was observed, and adult representation was limited. **Conclusions**: Cognitive functioning is significantly associated with multiple ICF domains in CP. Environmental Factors remain insufficiently addressed. Further research should consider CP heterogeneity and promote standardized assessments to support ICF-based intervention planning.

## 1. Introduction

Cerebral palsy (CP) is the most common cause of motor disability in childhood, with a prevalence of 1.6 per 1000 live births in high-income countries [1]. Moreover, CP is recognized as a highly heterogeneous condition, as motor symptoms are frequently accompanied by impairments in other domains such as cognition [2,3]. Among these cognitive impairments, intellectual disability affects approximately one in two children with CP [4], and the most frequently reported specific cognitive deficits include difficulties in visual perception [5] as well as impairments in both core and higher-order executive functions [6].

The combination of motor and cognitive challenges, among others, is thought to significantly limit participation and environmental exploration in individuals with CP [6]. Indeed, the systematic review by Shikako-Thomas et al. [7] demonstrated that children with CP engage in a narrower range of activities and exhibit lower social participation compared to their typically developing peers. To comprehensively understand the impact of these impairments on daily life, it is essential to analyze them within the framework of the International Classification of Functioning, Disability and Health (ICF) [8]. The ICF offers a biopsychosocial model that integrates not only impairments in body structures and functions but also participation and contextual factors, reinforcing the need to move beyond a biomedical model and adopt a broader, multidimensional perspective on health development. This perspective is crucial for guiding interventions aimed at improving quality of life and autonomy in individuals with any health condition. The ICF classification organizes information in a hierarchical structure, including different levels: components (Body Functions and Structures, Activities and Participation, Environmental Factors and Personal Factors), chapters within each component (e.g., d4 “Mobility” within Activities and Participation), and more specific second-level categories (e.g., d450 “Walking”). This multi-level framework allows for a comprehensive, standardized understanding of functioning across various domains of life.

Within this framework, ICF Core Sets have been developed to accurately describe various health conditions [9], including children, adolescents, and adults with CP [10,11]. For CP, the Core Sets emphasize the importance of the Activities and Participation and Environmental Factors components, with estimates suggesting that up to 70% of children’s well-being can be explained by categories related to these domains. Specifically, the ICF Core Set for children and youth with CP identifies 135 relevant categories distributed as follows: 5% related to Body Structures, 25% to Body Functions, 43% to Activities and Participation, and 27% to Environmental Factors [11], with a similar distribution observed in adults [10].

Additionally, although the ICF Core Sets for CP identify environmental factors as a key component, the scoping review by Santana et al. [12] highlights that these factors remain the least studied domain in the literature, with only 3% of included studies focusing on them. This discrepancy is particularly relevant given that environmental factors are potentially modifiable variables that can either facilitate or hinder participation and quality of life in people with CP. Along this line, Mei et al. [13] found that some environmental factors, such as negative attitudes of others, lack of support, or the availability of assistive devices, can significantly hinder the participation of children with CP. Thus, particular attention should be given to environmental factors such as access to and use of assistive products and technologies, personal support, and social attitudes, as well as the implementation of interventions tailored to the functional and cognitive needs of this population.

Despite the recognized importance of adopting a broader perspective, research on CP has historically focused predominantly on body structures and functions, particularly motor aspects, as highlighted in a recent scoping review by Santana et al. [12]. Consequently, most studies analyzing factors associated with activity, participation, and environmental components have prioritized motor functioning, consistently finding that motor skills are strong predictors of outcomes in these domains. The systematic review by Pashmdarfard et al. [14] concluded that gross and fine motor function is the main factor associated with participation in meaningful activities among children with CP (e.g., activities of daily living, play, and education), outweighing other personal and environmental factors.

Although the predictive role of motor skills is well established, there is growing evidence that particularly cognitive functioning plays a significant role in activities of daily living and participation. Importantly, participation is now understood to involve not only physical presence (“attendance”) but also the subjective experience of involvement, which depends on cognitive and motivational capacities; as a result, cognitive difficulties can limit participation in daily, social, educational, and leisure activities [14]. For instance, the narrative review by Bøttcher [15] detailed that cognitive impairments negatively affect participation and performance in social and educational contexts, in a process of mutual feedback. In line with this, recent studies underscore the dynamic interplay between cognitive, motor, and contextual factors: children with better cognitive and perceptual abilities are more likely to attend mainstream educational settings, which provide additional opportunities for social and motor development [16]. Notably, Andrade et al. [17] found that cognitive function, rather than motor impairment, was a significant predictor of school placement, highlighting the critical role of cognition in social participation and inclusion.

However, as Stadskleiv [3] points out, many studies rely solely on estimates of general cognitive functioning rather than conducting systematic, standardized assessments that evaluate not only overall cognitive performance but also specific cognitive domains such as attention, visual perceptual abilities, memory, and executive functions. This limitation hinders a full understanding of the true impact of cognition on functional outcomes. As highlighted by Pashmdarfard et al. [14], cognitive abilities, particularly those related to planning, problem-solving, attention, and memory, can significantly influence a child’s capacity to engage in and benefit from meaningful activities. Therefore, this review systematically examines, for the first time, the relationship between several cognitive domains assessed through standardized measures and the ICF components in individuals with CP.

In summary, the present systematic review aims to examine the role of specific cognitive domains in the ICF Activities and Participation and Environmental Factors components in people with CP. Specifically, this review seeks to address the following question: What role do cognitive functions play in the ICF components of Activities and Participation, as well as Environmental Factors, in people with CP? Undertaking this systematic review is essential not only for advancing theoretical understanding of the complex interplay between cognitive functioning, Activities and Participation, and Environmental Factors in CP but also for its significant clinical implications. Adopting an ICF-based, biopsychosocial approach is crucial for enabling multidimensional assessments and for planning personalized interventions.

## 2. Materials and Methods

This systematic review was registered in PROSPERO in August 2023: International Prospective Register of Systematic Reviews (CRD42023440241, https://www.crd.york.ac.uk/PROSPERO/view/CRD42023440241, accessed on 3 September 2025) and was conducted according to the Preferred Reporting Items for Systematic Reviews and Meta-Analysis (PRISMA) guideline [18]. 

### 2.1. Search Strategy

The search was conducted on 3 May 2023 and updated on 2 April 2025. The following databases were used: PubMed, PsycINFO, Central Register of Controlled Trials (CENTRAL), Cumulative Index to Nursing and Allied Health Literature (CINAHL), Education Resources Information Center (ERIC), and Web of Science (WOS). Text words related to CP, neuropsychological and cognitive assessment, and ICF-based activities, participation, and environmental factors were included. To select the optimal keywords, the second-level ICF categories included in the Comprehensive ICF Core Sets for children, youth, and adults with CP [10,11] were first reviewed to ensure that each component was represented by at least one search term. Based on this selection, an index term search was then conducted in both Medical Subject Headings (MeSH) and the APA Thesaurus of Psychological Index Terms to identify the most representative keywords for the search. The keywords used in the search are detailed in Table 1.

In each database, search terms were specified according to the platform’s indexing system: as Subject Headings in PsycINFO, CINAHL, and ERIC; as MeSH Terms in PubMed; and as Author Keywords in WOS and CENTRAL. The search was limited to studies conducted in humans and published from 2002, when the current version of the ICF was published by the World Health Organization (WHO) [19], through March 2025. Full search strings are provided in Supplementary Tables S1 and S2.

### 2.2. Eligibility of Studies

The studies were included if: (1) at least 70% of the sample had a confirmed diagnosis of CP. In the case of studies with a lower percentage, they were only considered if they presented the results separately for the CP group; (2) the study had to assess CP participants using standardized measures for at least one of the cognitive domains listed as keywords above; (3) the study had to consider aspects of the ICF Activities and Participation and/or Environmental Factors components. These components had to be measured using standardized tests. However, in the case of Environmental Factors, given the scarcity of available standardized measures, it was considered sufficient for inclusion the studies that systematically collected environmental factors (4) the studies had to statistically analyze the relationship between cognitive and ICF variables. In contrast, non-peer-reviewed studies, review articles, articles published in languages other than English or Spanish, and articles whose full text was not available were not included in the present systematic review.

### 2.3. Procedure

Data were managed using the web-based platform PICO Portal (https://picoportal.org/), which facilitated duplicate identification and article selection. Two independent reviewers screened the titles and abstracts of all identified articles. Full texts of studies that appeared to meet the eligibility criteria were then assessed by the same two independent reviewers. Studies were included in the analysis only if both reviewers agreed that the inclusion criteria were met. Any disagreements regarding study eligibility were resolved through discussion; if consensus could not be reached, a third reviewer was consulted to make the final decision.

### 2.4. Data Extraction

Data were extracted independently by one reviewer using a predefined form. A second reviewer checked the data extraction for each individual study and recorded any changes in the same form. Any disagreements between reviewers were resolved through discussion. If consensus could not be reached, a third reviewer made the final decision. The following information was considered relevant for extraction: (1) Study (article reference, type and aim/s); (2) Sample characteristics (inclusion criteria, number of participants diagnosed with CP, age, sex, type of CP, pattern of involvement and functional measures); (3) Cognitive assessment (cognitive domain/s assessed, instrument/s used and their characteristics); (4) ICF components assessment (ICF component, chapter and second-level categories, instrument/s used and their characteristics). In the case of therapeutic interventions, the name of the intervention as well as the type and dosage applied were recorded; (5) Results (statistical analyses applied and results obtained).

### 2.5. Quality Assessment

A qualitative evaluation of the studies was performed according to the Oxford Centre for Evidence-Based Medicine (OCEBM) 2011 [20], as performed in previous systematic reviews of CP [21,22]. Level of evidence (LOE) was graded from 1 (high) to 5 (low): level 1, systematic review of randomized controlled trials (RCTs) or n-of-trials; level 2, RCTs or observational study with a dramatic effect; level 3, non-randomized controlled cohort/follow-up study; level 4, case series, case–control study or a historically controlled study; and level 5, mechanism-based reasoning.

## 3. Results

**Figure 1:**
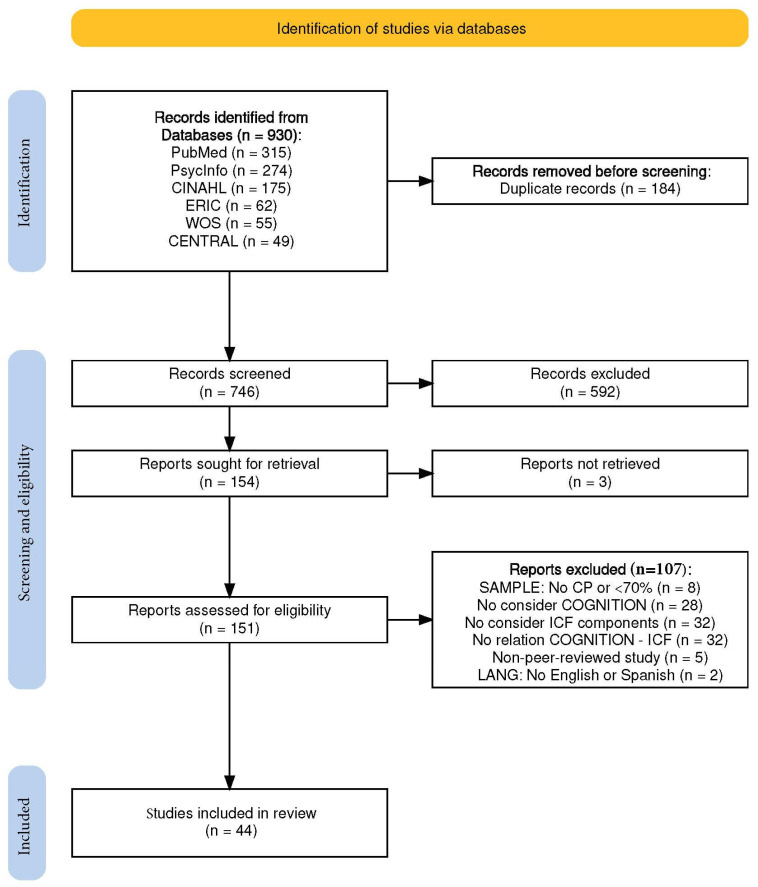
PRISMA Flow diagram of the study selection process.

The results of the search strategy are shown in Figure 1, produced using the PRISMA 2020 R package (version 1.1.2) [23]. The searches returned 930 articles: 315 from PubMed, 274 from PsycINFO, 175 from CINAHL, 62 from ERIC, 55 from WOS, and 49 from CENTRAL. Removing duplicates resulted in 746 articles, and screening titles and abstracts resulted in 154 full-text articles for review. Finally, 44 articles were included. Twenty-three percent of the included studies (10/44) correspond to an OCEBM LOE 2 (RCTs or observational study with a dramatic effect), 16% (7/44) to level 3 (non-randomized controlled cohort/follow-up study), and 61% (27/44) to level 4 (case series, case–control study or a historically controlled study). Details of each of the 44 studies included in this review are provided in Supplementary Tables S3–S5.

### 3.1. Sample Characteristics

The combined sample from the 44 studies included a total of 3056 individuals with CP, ranging in age from 1 to 29 years. Of the 44 studies, 43 included children and/or adolescents (<20 years), while only one study included adolescents and young adults up to 29 years [24]. Delving deeper, the distributions in terms of mean age were as follows: 0–6 years: 13 studies, 7–12 years: 26 studies, and 13–18 years: 3 studies. Among the studies that reported sex, 1241 participants (41.2%) were female.

Regarding CP type, 32 (72.7%) studies provided this information. Most participants (*n* = 1729, 82.5%) were diagnosed with spastic CP, followed at a considerable distance by dyskinetic (*n* = 131, 6.3%), mixed (*n* = 88, 4.2%), ataxic (*n* = 51, 2.4%), and other (*n* = 78, 3.7%). Despite dyskinetic being the second most common CP type, only 12 out of 44 studies (27.3%) included subjects with this type of CP in their samples. Concerning the distribution pattern of CP, bilateral was the most frequently reported, accounting for 1463 cases (68% of those with available data). The Gross Motor Function Classification System (GMFCS) level of participants was reported in 31 studies, covering 2604 participants. The majority had mild to moderate motor impairment (GMFCS I to III), indicating the ability to walk independently or with assistive devices. Nineteen out of 44 studies (43.2%) reported including subjects with severe CP (GMFCS IV and V).

### 3.2. Distribution of ICF Chapters in Included Studies

**Figure 2:**
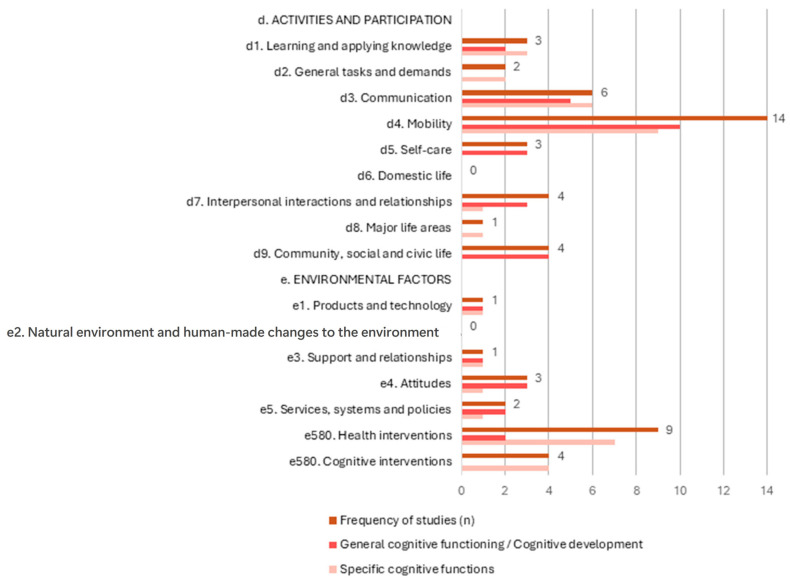
Frequency of ICF components and chapters analyzed by included studies, and cognitive function assessed.

Figure 2 illustrates the frequency with which each ICF component was addressed in the 44 included studies. The results are organized by ICF chapters and presented first as total frequencies (in brown color), followed by a breakdown distinguishing whether the studies focused on general cognitive functioning or cognitive development (in red), or on specific cognitive functions (in pink). The most frequently analyzed chapter is d4. Mobility, which is addressed in 14 out of 44 studies (31.8%), is the component whose relationship with cognitive function has been studied the most. Moreover, it has been the most frequently addressed ICF chapter in articles with samples with a mean age of 0–6 years, and the second most in those with a mean age of 7–12 years. These findings suggest a strong research focus on how cognitive abilities relate to physical movement and vice versa, especially in early and middle childhood populations. Following this, health interventions, which fall under chapter e5. Services, systems, and policies constitute the second most frequently studied ICF chapter, appearing in nine studies (20.5%). Notably, it was the most often examined among individuals with mean ages of 7–12 and was also studied in those aged 13–18, reflecting the significant role that structured services and systemic support play in cognitive development during these age ranges. The third most frequent is d3. Communication was analyzed in six studies (13.6%), indicating interest in the relationship between cognitive function and receiving and/or producing messages. Other chapters referenced in fewer than five studies can be seen in Figure 2, pointing to a moderate focus on social functioning and formal intervention strategies in relation to cognitive performance.

At the opposite end, no studies were found that examined the relationship between cognitive functioning and chapter d6. Domestic life or e2. Natural environment and human-made changes to the environment, indicating that these domains remain entirely unexplored within the current body of cognitive research on CP.

The following section offers a comprehensive review of the specific findings from the included studies, organized by ICF components and chapters. First, Activities and Participation (d) domains will be presented, followed by Environmental Factors (e). In both components, the results identified for each chapter within the domain will be presented separately.

Furthermore, the analysis will pay particular attention to key variables such as age, type of CP, and level of motor severity. Special emphasis will be placed on less frequent and more severe cases, including dyskinetic CP and individuals classified at GMFCS levels IV and V. Whenever a pattern is identified, the most significant findings related to these variables will be discussed in detail. This approach aims to provide a clearer interpretation of the results and to highlight factors that may be underrepresented across the studies analyzed. Finally, each ICF chapter will conclude with a set of concise summary statements to highlight the principal conclusions.

### 3.3. Activities and Participation

#### 3.3.1. Learning and Applying Knowledge (d1)

This ICF chapter of Learning and applying knowledge (d1) focuses on the individual’s ability to acquire and apply knowledge and skills, encompassing basic learning, problem-solving, reading, writing, and decision-making across daily and academic contexts. Three articles from the same research group explore this chapter with a specific focus on literacy and its relationship with general intellectual function and language. The studies examined a sample of children with a mean age of 6 years, primarily diagnosed with spastic CP and ranging from mild intellectual disability to average or above-average intelligence, with OCEBM LOE levels of 3 (*n* = 1) and 4 (*n* = 2). Detailed demographic characteristics, as well as the cognitive and ICF instruments used, are provided in Table 2.

Within the scholarly context, the amount and time of instruction in reading precursors (e.g., auditory perception, synthesis, and analysis) were the only factors associated with emergent literacy skills such as vocabulary and syntactic language skills. These factors, as well as time dedicated to storybook reading, were also associated with the level of general intellectual functioning. Specifically, Peeters et al. [25] reported that less instructional time dedicated to literacy activities and fewer specific reading precursors being taught were linked to lower levels in language and intelligence functions.

A similar pattern emerged when addressing the activities, materials, interests, and expectations related to literacy in the home setting (home literacy environment). Intelligence showed a significant positive correlation with two of the 13 home literacy factors examined: child engagement in word orientation activities (e.g., reading aloud, pointing at words) and parental involvement in literacy mediation (e.g., engaging the child in the parent’s own reading, playing rhyme games) [26]. Vocabulary also demonstrated a significant positive relationship with these home literacy activities, although this finding was only consistent in one of the two papers that investigated the association [26,27].

**Table 2:** Learning and Applying Knowledge (d1).

ICF		Cognitive Assessment	Demographic Data	Main Results *
**ICF chapter**	**ICF second level** *Assessment*	**Cognitive domain** *Instrument*	n Age range (years:months) n females n type CP n pattern CP Motor ability	**+ (significative)** **− (significative)** **n.s.** Author (year)—LOE
**d1 Learning and applying knowledge**	**d166 Reading** *Five parent questionnaires regarding Home Literacy Variables* *Questionnaire about emergent literacy activities*	**General intellectual functioning** *Raven Coloured Progressive Matrices (RCPM)*	92 5:0–6:3 years, 40 unk 36 females 78 spastic, 3 ataxic, 11 mixed 16 unilateral, 66 bilateral, 10 unk Mobility ability unk	**+ (significative)** Peeters et al. (2009) [26]—LOE 4 Peeters et al. (2011) [25]—LOE 4 **n.s.** Peeters et al. (2009) [26]—LOE 4 Peeters et al. (2011) [25]—LOE 4
	**d166 Reading** *Five parent questionnaires regarding Home Literacy Variables* *Four self-administrated parent questionnaires* *Questionnaire about emergent literacy activities*	**Language** *Dutch Language Proficiency Test* *Dutch Specific Language Impairment (SLI)* *Peabody Picture Vocabulary Test—3rd edition (PPVT-III)* *Reading Technology Test, Shortened Version*	127 5:0–7:0 years, 40 unk 57 females 112 spastic, 4 ataxic, 11 mixed 21 unilateral, 91 bilateral, 15 unk Mobility ability unk	**+ (significative)** Peeters et al. (2009) [27]—LOE 3 Peeters et al. (2011) [25]—LOE 4 **n.s.** Peeters et al. (2009) [26]—LOE 4 Peeters et al. (2009) [27]—LOE 3 Peeters et al. (2011) [25]—LOE 4

Abbreviations: − (significative), negative tendency with significative results; + (significative), positive tendency with significative results; CP, cerebral palsy; ICF, International Classification of Functioning, Disability and Health; LOE, level of evidence; n.s., no significative results; unk, unknown; italic format, measures used; *, detailed results are presented in Supplementary Table S3.

Further analysis of the language domain, based on the findings of Peeters et al. [27], indicated that child word/story (e.g., commenting on the story, asking questions) orientation activities and parent literacy mediation were significantly associated with syntactic skills, letter knowledge, and word recognition. Child writing experiences (e.g., use of writing and drawing material) and book orientation activities (e.g., flip the page, handle the book) were significantly associated with syntactic skills, while parent leisure activities (e.g., play with the kid) were significantly associated both with letter knowledge and word recognition. Although these results suggest a relationship between the home literacy environment and early emergent literacy skills (including language and reading abilities), hierarchical multiple regression analyses revealed that this association is primarily explained by the training of specific reading precursors. Consequently, the authors recommend that parents prioritize fostering these foundational skills to support the development of emergent literacy.

As a summary, both school-based instruction and family involvement in the teaching of reading precursors support the development of emergent literacy in children with CP. These findings highlight the need for multidisciplinary teams working with children with CP to coordinate strategies with schools and families, particularly at the stage of literacy acquisition, in order to develop individualized interventions that maximize learning outcomes.

#### 3.3.2. General Tasks and Demands (d2)

The General tasks and demands (d2) chapter covers the execution of single or complex tasks, including initiating, organizing, and managing responsibilities and routines in everyday life, as well as handling stress and adapting to change. This domain is explored in two articles included in this review, both with an OCEBM LOE of 4. These studies examined, in children and adolescents aged 8 to 17 years with mild unilateral CP (GMFCS levels I–II), the relationship between this ICF domain and executive functioning (*n* = 46) [28], as well as visual perception (*n* = 101) [29] (Table 3).

Using the Behaviour Rating Inventory of Executive Function (BRIEF), a questionnaire completed by parents and teachers that evaluates children’s ability to manage tasks and self-regulate in everyday contexts, Whittingham et al. [28] found that poorer performance in executive functioning was associated with higher scores on the Behavioural Regulation Index (parent report), which reflects difficulties in organizing and completing task sequences within the home environment.

Similarly, James et al. [29] found significant positive correlations between overall performance on the Test of Visual Perceptual Skills (TVPS) and the General tasks and demands chapter, with two TVPS subtests and a motor variable related to the ability of the dominant upper limb emerging as the strongest predictors in their model. Notably, the TVPS Visual Sequential Memory subtest was identified as the most influential contributor, suggesting that better visual processing skills are associated with greater competence in managing daily routines, even in individuals with mild CP.

These studies collectively emphasize that both executive and visual–perceptual skills play a key role in supporting daily task performance in youth with mild unilateral CP. Identifying difficulties with daily routines linked to cognitive functions supports the integration of neuropsychological assessment into therapeutic planning, enabling the design of tailored supports that enhance autonomy in activities of daily living.

**Table 3:** General Tasks and Demands (d2).

ICF		Cognitive Assessment	Demographic Data	Main Results *
**ICF chapter**	**ICF second level** *Assessment*	**Cognitive domain** *Instrument*	n Age range (years:months) n females n type CP n pattern CP Motor ability	**+ (significative)** **− (significative)** **n.s.** Author (year)—LOE
**d2 General tasks and demands**	**d230 Carrying out daily routine** *Behaviour Rating Inventory of Executive Function (BRIEF)*	**Executive functions** *Delis–Kaplan Executive Function System (D-KEFS)* *Rey Complex Figure Test (RCFT)* *Test of Everyday Attention for Children (TEA-Ch)* *Wechsler Intelligence Scale for Children—4th edition (WISC-IV)*	46 8:0–16:0 years 21 females CP type unk 46 unilateral GMFCS: 35 I, 11 II MACS: 6 I, 40 II	**− (significative)** Whittingham et al. (2014) [28]—LOE 4
	**d230 Carrying out daily routine** *Assessment of Motor and Process Skills–7th edition (AMPS)*	**Visual perception** *Test of Visual Perceptual Skills (Non-Motor)—3rd edition (TVPS-3)*	101 8:0–17:0 years 50 females 101 spastic 101 unilateral GMFCS: 45 I, 56 II MACS: 24 I, 76 II, 1 III	**+ (significative)** James et al. (2015) [29]—LOE 4

Abbreviations: − (significative), negative tendency with significative results; + (significative), positive tendency with significative results; CP, cerebral palsy; GMFCS, Gross Motor Function Classification System; ICF, International Classification of Functioning, Disability and Health; LOE, level of evidence; MACS, Manual Ability Classification System; n.s., no significative results; unk, unknown; italic format, measures used; *, detailed results are presented in Supplementary Table S3.

#### 3.3.3. Communication (d3)

This ICF chapter addresses how individuals receive, produce, and exchange information through language, signs, symbols, and technologies, including the use of communication devices. Among the cognitive domains, language was the most frequently assessed in relation to the Communication (d3) chapter of the ICF (Table 4).

Six studies explored the relationship between language abilities and communication outcomes in samples with predominant spastic CP type, yielding mixed results. Some reported non-significant associations (*n* = 52) [30,31,32]. Interestingly, after controlling for mental age (*n* = 10), Holck et al. [31] reported a significant correlation indicating that children with a higher comprehension of literal information performed worse in pragmatic abilities. Other studies found that higher language performance was significantly related to better communication skills (*n* = 137) [33,34,35]. Across all studies, Pennington et al. [35] provided the highest level of evidence (OCEBM LOE 3) and included the youngest sample, with a mean age of 28.6 months. The other studies were classified as OCEBM LOE 4 and included children and adolescents up to 18 years old.

Notably, when comparing studies using the same language measures (Test for Reception of Grammar Version 2 and Peabody Picture Vocabulary Test—Fourth Edition), Koopmans et al. [32] and Nordberg et al. [34] reported different findings. The study that identifies a significant relationship included a less severe sample (no cases with GMFCS V and all participants had intelligible speech), suggesting that differences in sample characteristics and comorbidities may influence results [34].

In this regard, it is worth noting that the Communication (d3) chapter has been one of the most frequently explored ICF chapters in relation to the dyskinetic CP and GMFCS levels IV and V. Studies with larger samples of individuals with dyskinetic CP (*n* = 25) [33,34,35] and those classified at GMFCS levels IV–V (*n* = 45) [33,34,35] tended to report significant associations between language performance and communication skills. Conversely, studies that did not find significant associations generally included fewer participants with dyskinetic CP (*n* = 2) [30] and GMFCS IV–V (*n* = 10) [32]. This pattern suggests that the representation of more severe motor impairment and specific CP subtypes within samples may influence the detection of relationships between language abilities and communication outcomes.

General intellectual functioning was the other focus in five studies, presenting a wide age range among participants (Table 4). Participants classified at lower severity levels on the Communication Function Classification System (CFCS) or Functional Communication Classification System (FCCS) [32,33,35] tended to demonstrate higher intellectual functioning scores. Studies using the Raven’s Coloured Progressive Matrices (RCPM) as a measure of general intellectual functioning found no significant associations with overall communication measures except for a positive correlation between sentence length on the Bus Story Test and RCPM scores [30,34].

Considering that significant associations are reported in three of the four studies including participants with GMFCS IV–V (*n* = 52) [32,33,35], and that these studies also involved larger sample sizes, it can be observed that the association between general intellectual functioning and communication follows a similar pattern to that described between the language function and this ICF chapter, at least in terms of motor capacity. Regarding the dyskinetic type, no conclusions can be drawn, since the number of studies supporting a significant association between general intellectual functioning and communication [33,35] is equal to those reporting non-significant associations [30,34], and the sample sizes do not allow for establishing a clear trend.

**Table 4:** Communication (d3).

ICF		Cognitive Assessment	Demographic Data	Main Results *
**ICF chapter**	**ICF second level** *Assessment*	**Cognitive domain** *Instrument*	n Age range (years:months) n females n type CP n pattern CP Motor ability	**+ (significative)** **− (significative)** **n.s.** Author (year)—LOE
**d3 Communication**	**d310–d329 Communicating—receiving** **d330–d349 Communicating—producing** **d350–d369 Conversation and use of communication devices and techniques** *Communication Function Classification System (CFCS)* *Functional Communication Classification Scale (FCCS)* **d330 Speaking** *Bus Story Test (BST)* *Narrative Assessment Profile (NAP)* **d331 Non-speech vocal expression** **d335 Producing nonverbal messages** *Material from Dahlgren, Sandberg, and Hjelmquist 1996*	**General intellectual functioning** *Leiter International Performance Scale—Revised (Leiter-R)* *Mullen Scales of Early Learning (MSEL)* *Raven’s Coloured Progressive Matrices (RCPM)*	179 2:0–18:1 years 68 females 131 spastic, 27 dyskinetic, 9 ataxic, 3 mixed, 2 other, 7 unk 37 unilateral, 94 bilateral, 48 unk GMFCS: 55 I, 35 II, 27 III, 24 IV, 31 V, 7 unk MACS: 30 I, 66 II, 24 III, 22 IV, 15 V, 22 unk	**− (significative)** Asano et al. (2023) [33]—LOE 4 Koopmans et al. (2022) [32]—LOE 4 Nordberg et al. (2015) [34]—LOE 4 Pennington et al. (2020) [35]—LOE 3 **n.s.** Falkman et al. (2002) [30]—LOE 4 Nordberg et al. (2015) [34]—LOE 4
	**d310–d329 Communicating—receiving** **d330–d349 Communicating—producing** **d350–d369 Conversation and use of communication devices and techniques** *Communication Function Classification System (CFCS)* *Functional Communication Classification Scale (FCCS)* **d310–d329 Communicating—receiving** *Children’s Communication Checklist (CCC)* **d330 Speaking** *Bus Story Test (BST)* *Narrative Assessment Profile (NAP)* **d331 Non-speech vocal expression** **d335 Producing nonverbal messages** *Material from Dahlgren, Sandberg, and Hjelmquist 1996*	**Language** *MacArthur Communicative Development Inventory (MCDI)* *Material from Bishop and Adams (1992; translated and adapted to Swedish by the authors)* *Peabody Picture Vocabulary Test—4th edition (PPVT-4)* *Picture Vocabulary Test (PVT)* *Preschool Language Scales—4th edition (PLS-4)* *Språkligt Impressivt Test (SIT)* *Syntactic acceptability* *Test for Auditory Comprehension of Language—4th edition (TACL-4)* *Test for Reception of Grammar—2nd edition (TROG-2)*	189 2:0–18:1 years 71 females 141 spastic, 27 dyskinetic, 9 ataxic, 3 mixed, 2 other, 7 unk 47 unilateral, 94 bilateral, 48 unk GMFCS: 55 I, 35 II, 27 III, 24 IV, 31 V, 17 unk MACS: 30 I, 66 II, 24 III, 22 IV, 15 V, 32 unk	**+ (significative)** Asano et al. (2023) [33]—LOE 4 Nordberg et al. (2015) [34]—LOE 4 Pennington et al. (2020) [35]—LOE 3 **− (significative)** Holck et al. (2010) [31]—LOE 4 **n.s.** Falkman et al. (2002) [30]—LOE 4 Holck et al. (2010) [31]—LOE 4 Koopmans et al. (2022) [32]—LOE 4
	**d330 Speaking** *Bus Story Test (BST)* *Narrative Assessment Profile (NAP)*	**Executive functions** *Corsi block-tapping test (CB)* *Wechsler Intelligence Scale for Children—3rd edition (WISC-III)*	15 9:2–12:9 years 7 females 10 spastic, 2 dyskinetic, 3 ataxic 8 unilateral, 2 bilateral, 5 unk GMFCS: 9 I, 1 II, 2 III, 3 IV	**n.s.** Nordberg et al. (2015) [34]—LOE 4
	**d330 Speaking** *Bus Story Test (BST)* *Narrative Assessment Profile (NAP)*	**Memory** *Corsi block-tapping test (CB):* forward *Wechsler Intelligence Scale for Children—3rd edition (WISC-III)*	15 9:2–12:9 years 7 females 10 spastic, 2 dyskinetic, 3 ataxic 8 unilateral, 2 bilateral, 5 unk GMFCS: 9 I, 1 II, 2 III, 3 IV	**+ (significative)** Nordberg et al. (2015) [34]—LOE 4 **n.s.** Nordberg et al. (2015) [34]—LOE 4
	**d330 Speaking** *Bus Story Test (BST)*	**Social cognition** *False belief items of 2 story tests: “Kiki and the cat” and “Birthday puppy”*	15 9:2–12:9 years 7 females 10 spastic, 2 dyskinetic, 3 ataxic 8 unilateral, 2 bilateral, 5 unk GMFCS: 9 I, 1 II, 2 III, 3 IV	**+ (significative)** Nordberg et al. (2015) [34]—LOE 4

Abbreviations: − (significative), negative tendency with significative results; + (significative), positive tendency with significative results; CP, cerebral palsy; GMFCS, Gross Motor Function Classification System; ICF, International Classification of Functioning, Disability and Health; LOE, level of evidence; MACS, Manual Ability Classification System; n.s., no significative results; unk, unknown; italic format, measures used; *, detailed results are presented in Supplementary Table S3.

Additional cognitive aspects have also been explored in relation to communication (Table 4). Nordberg et al. [34] examined memory span, working memory (executive functions), and social cognition in relation to narrative ability. Results showed that auditory memory span, but not visuospatial span or working memory (auditory and visual), was significantly correlated with retelling ability in 15 children aged 9 to 12 years, indicating that better auditory short-term memory supports narrative skills. In terms of social cognition, a significant relationship was observed between Theory of Mind (second-order beliefs) and story retelling ability, suggesting that better social cognition is related to better narrative abilities.

In summary, while definitive conclusions cannot be drawn, stronger language and general intellectual functioning may be associated with better communication skills when considering the studies with the largest samples and/or higher level of evidence of the set. Additionally, while some memory and social cognition components are related to communication, the associations are not always straightforward and may vary depending on the specific cognitive domain. These results underscore the value of early assessment of both general cognitive and specific language abilities to guide individualized speech–language interventions or augmentative communication supports, thereby promoting children’s social and educational participation.

#### 3.3.4. Mobility (d4)

The ICF Mobility chapter (d4) encompasses the ability to move and change body position or location, including walking, climbing, transferring, and using transportation or mobility aids to navigate the environment. A significant number of articles (*n* = 14) in this review address this ICF chapter. And particularly, it has been the most studied in samples comprising dyskinetic CP type, and GMFCS IV–V.

First, articles that study the relationship with general intellectual functioning will be presented, followed by those that address specific cognitive domains.

Ten studies examined the relationship between this domain and cognitive functioning or development in children with CP (Table 5). Most participants had a spastic type of CP, and all levels of gross motor function and manual ability were represented.

Eight studies assessed the link between gross motor skills and cognitive functioning in children and adolescents up to 18 years old, but the findings were inconsistent. Three studies (*n* = 775) reported significant associations, indicating that better motor abilities were related to higher scores in general intellectual functioning or cognitive development [33,36,37]. In contrast, five studies with a combined smaller sample (*n* = 448), including two with higher levels of evidence (OCEBM LOE 3), did not find significant associations [38,39,40,41,42]. All levels of motor severity were included, with GMFCS level V being highly represented. In this regard, in the overall sample of the eight studies, there appears to be no association between these variables in individuals with GMFCS Level I. However, in the other gross motor levels (II to V), there was a marked tendency toward significant results, notably in the GMFCS V level. This is consistent with the fact that of the four studies that included dyskinetic CP (*n* = 77) [33,36,37,42], three were the ones exhibiting significant results (*n* = 63) [33,36,37]. However, it should be noted that not all studies provide data regarding motor ability and CP type.

When manual ability was analyzed, greater consistency emerged, particularly when considering the distribution pattern (unilateral vs. bilateral). Three studies (*n* = 149) found that a higher intellectual level was associated with better manual ability, especially in individuals with bilateral CP, with one study controlling for chronological age [33,43,44]. Only one study (*n* = 49) reported no significant relationship with bilateral CP [41]. In this case, the milder levels of the Manual Ability Classification System (MACS) (I–II) were the most represented, although data are available from only two of the studies that reported significant associations [33,43].

Research exploring specific cognitive domains and their relationship with mobility in CP has focused primarily on language, visual perception, attention, processing speed and executive functions, with most studies presenting moderate to low levels of evidence and samples composed mainly by participants with spastic CP, with only two studies including individuals with dyskinetic type [33,45], but on a negligible proportion of their sample (*n* = 4 out of 85) (Table 5).

Regarding language and verbal functioning, six studies involving a total of 253 children and adolescents examined this association [33,38,40,41,44,46]. Three of these studies reported significant relationships, identifying that children with better gross and manual motor abilities also exhibited higher emergent literacy, expressive, and comprehension skills [38,41,46]. Regarding the level of impairment in terms of gross motor function, when the results were significant, all GMFCS levels were represented, with no particular level standing out. However, not all studies provided this data. Regarding manual ability, only the study by Asano et al. [33] reported results, with level II being the most represented and no cases at level V. In addition, three studies explored the relationship between visual perception and nonverbal functioning with both gross motor function and manual ability [29,44,45]. Two studies identified associations, suggesting that better performance in visual perception is related to higher motor ability [29,44] in a sample with mild GMFCS and MACS levels [29]. However, while these associations were found, visual perception was not a significant predictor in multivariable models [29]. Additionally, when using different statistical approaches, no clear differences emerged when comparing groups according to motor severity in a sample without GMFCS level V [45].

Within the domain of attention, one study involving 45 participants with a mean age of approximately 12 years and classified at MACS levels I–II found significant correlations between hand/arm use and attentional performance, suggesting that better fine motor skills are associated with superior performance in attention [47]. For processing speed, the only available study did not find differences across gross motor severity levels in the 15 children assessed [40]. In the domain of executive functions, working memory outcomes were not related to gross motor functioning when comparing groups of different motor severity [40,45], although some differences were observed in specific tasks between levels I and III/IV [45].

Overall, while the association between intellectual functioning and gross motor skills remains ambiguous, a clearer pattern links higher cognitive capacity with better manual motor skills, particularly in bilateral CP. Language and visual perception emerge as the cognitive processes most related to gross motor abilities, although the findings are not entirely conclusive. For fine motor skills, visual perception, and attention appear to be the cognitive domains most closely linked. Similar to what has been described for general intellectual functioning, when significant associations are observed, they consistently indicate that better performance in these cognitive domains is associated with higher mobility outcomes. These results underscore the necessity of screening key cognitive functions in children with CP as soon as possible, particularly those with more severe motor impairments, to identify when a comprehensive neuropsychological assessment should be conducted. This approach would enable professionals to design integrated interventions that address not only motor challenges, but also related cognitive and educational needs, thereby ensuring a more holistic support.

**Table 5:** Mobility (d4).

ICF		Cognitive Assessment	Demographic Data	Main Results *
**ICF chapter**	**ICF second level** *Assessment*	**Cognitive domain** *Instrument*	n Age range (years:months) n females n type CP n pattern CP Motor ability	**+ (significative)** **− (significative)** **? (significative)** **n.s.** Author (year)—LOE
**d4 Mobility (Gross motor functions)**	**d410–d429 Changing and maintaining body position** **d430–d449 Carrying, moving, and handling objects** **d450–d469 Walking and moving** *Bayley Infant Development Screening Test—2nd edition (BSID-II)* *Gross Motor Function Classification System (GMFCS)* *Gross Motor Function Classification System Family Report Questionnaire (GMFCS-FR)* *Gross Motor Function Classification System, Expanded and Revised (GMFCS E&R)* *Gross Motor Function Measure (GMFM)* *Pediatric Evaluation of Disability Inventory (PEDI)* *Selective Motor Control (SMC)*	**General intelligence functioning** *Raven’s Coloured Progressive Matrices (RCPM)* *Wechsler Intelligence Scale for Children—4th edition (WISC-IV)* *Wechsler Preschool and Primary Scale of Intelligence (WPPSI)* *Wechsler Intelligence Scale for Children—Revised (WISC-R)* *Comprehensive Developmental Inventory for Infants and Toddlers (CDIIT)* **Cognitive development** *Bayley Infant Development Screening Test—2nd edition (BSID-II)* *Pediatric Evaluation of Disability Inventory—Computer Adaptive Test, Speedy Version (PEDI-CAT)*	1223 0:1–18:0 years, 49 unk 503 females 760 spastic, 77 dyskinetic, 25 ataxic, 63 mixed, 30 other, 268 unk 183 unilateral, 805 bilateral, 232 unk GMFCS: 197 I, 198 II, 205 III, 212 IV, 294 V, 117 unk MACS: 48 I, 63 II, 20 III, 9 IV, 11 V, 1072 unk	**+ (significative)** Asano et al. (2023) [33]—LOE 4 Dalvand et al. (2012) [36]—LOE 4 Song (2013) [37]—LOE 4 **n.s.** Chen et al. (2013) [38]—LOE 2 Fontes et al. (2025) [39]—LOE 4 Muriel et al. (2014) [40]—LOE 4 Peeters et al. (2009) [41]—LOE 4 Smits et al. (2011) [42]—LOE 3
	**d410–d429 Changing and maintaining body position** **d450–d469 Walking and moving** *Gross Motor Function Classification System (GMFCS)* *Gross Motor Limitation Scale* *Selective Motor Control (SMC)*	**Language** *Dutch Language Proficiency Test* *Peabody Picture Vocabulary Test—3rd edition (PPVT-III)* *Reynell Developmental Language Scale—Revised (RDLS-R)* *Comprehensive Developmental Inventory for Infants and Toddlers (CDIIT)* *Bo Ege Test Verbal Language Development Scale* *Wechsler Intelligence Scale for Children—4th edition (WISC-IV)* *Picture Vocabulary Test (PVT)*	223 1:0–18:0 years, 49 unk 85 females 127 spastic, 1 dyskinetic, 3 ataxic, 92 unk 25 unilateral, 154 bilateral, 44 unk GMFCS: 39 I, 32 II, 33 III, 21 IV, 20 V, 12 I–II, 17 IV–V, 49 unk MACS: 15 I, 23 II, 6 III, 1 IV, 178 unk	**+ (significative)** Chen et al. (2013) [38]—LOE 3 Peeters et al. (2009) [41]—LOE 4 Pirila et al. (2007) [46]—LOE 4 **n.s.** Asano et al. (2023) [33]—LOE 4 Muriel et al. (2014) [40]—LOE 4
	**d410–d429 Changing and maintaining body position** **d450–d469 Walking and moving** *Gross Motor Function Classification System (GMFCS)*	**Executive functions** *Wechsler Intelligence Scale for Children—4th edition (WISC-IV)* *Corsi Block-tapping test (CBT)* *Walking Corsi Test (WalCT)*	55 5:0–17:0 years 23 females 51 spastic, 3 dyskinetic, 1 ataxic 11 unilateral, 41 bilateral, 3 unk GMFCS: 23 I, 16 II, 2 III, 3 V, 11 III–IV	**? (significative)** Bartonek et al. (2021) [45]—LOE 4 **n.s.** Bartonek et al. (2021) [45]—LOE 4 Muriel et al. (2014) [40]—LOE 4
	**d410–d429 Changing and maintaining body position** **d450–d469 Walking and moving** *Gross Motor Function Classification System (GMFCS)* **d410–d429 Changing and maintaining body position** **d430–d449 Carrying, moving, and handling objects** *Assessment of Motor and Process Skills—7th edition (AMPS)*	**Visual perception** *Raven’s Coloured Progressive Matrices (RCPM)* *Test of Visual Perceptual Skills (Non-Motor)—3rd edition (TVPS-3)*	141 5:0–17:0 years 66 females 138 spastic, 3 dyskinetic 105 unilateral, 33 bilateral, 3 unk GMFCS: 62 I, 68 II, 11 III-IV MACS: 24 I, 76 II, 1 III, 40 unk	**+ (significative)** James et al. (2015) [29]—LOE 4 **n.s.** Bartonek et al. (2021) [45]—LOE 4 James et al. (2015) [29]—LOE 4
	**d410–d429 Changing and maintaining body position** **d450–d469 Walking and moving** *Gross Motor Function Classification System (GMFCS)*	**Processing speed** *Wechsler Intelligence Scale for Children—4th edition (WISC-IV)*	15 7:0–14:0 years 7 females 14 spastic, 1 ataxic 7 unilateral, 8 bilateral GMFCS: 6 I, 4 II, 2 III, 3 V	**n.s.** Muriel et al. (2014) [40]—LOE 4
**d4 Mobility (Manual ability)**	**d440 Fine hand use** **d430–d449 Carrying, moving, and handling objects** *Both Hands Assessment (BoHA)* *Computerised Peg Moving Task (CPMT)* *Kids Assisting Hand Assessment (AHA)* *Manual Ability Classification System (MACS)*	**General intelligence functioning** *Raven’s Coloured Progressive Matrices (RCPM)*	198 4:0–18:0 years, 49 unk 71 females 187 spastic, 5 dyskinetic, 5 ataxic, 1 mixed 63 unilateral, 124 bilateral, 11 unk GMFCS: 57 I, 30 II, 21 III, 11 IV, 79 unk MACS: 45 I, 51 II, 22 III, 1 IV, 79 unk	**+ (significative)** Asano et al. (2023) [33]—LOE 4 Burgess et al. (2021) [43]—LOE 4 Dellatolas et al. (2005) [44]—LOE 4 **n.s.** Burgess et al. (2021) [43]—LOE 4 Dellatolas et al. (2005) [44]—LOE 4 Peeters et al. (2009) [41]—LOE 4
	**d430–d449 Carrying, moving, and handling objects** *Computerised Peg Moving Task (CPMT)* *Manual Ability Classification System (MACS)*	**Language** *Dutch Language Proficiency Test* *Neuropsychological battery* *Peabody Picture Vocabulary Test—3rd edition (PPVT-III)* *Picture Vocabulary Test (PVT)*	124 4:0–18:0 years, 49 unk 45 females 120 spastic, 1 dyskinetic, 2 ataxic, 1 mixed 33 unilateral, 87 bilateral, 4 unk GMFCS: 13 I, 12 II, 13 III, 7 IV, 79 unk MACS: 15 I, 23 II, 6 III, 1 IV, 79 unk	**+ (significative)** Peeters et al. (2009) [41]—LOE 4 **n.s.** Asano et al. (2023) [33]—LOE 4 Dellatolas et al. (2005) [44]—LOE 4
	**d445 Hand and arm use** *Object hit and avoid task using the Kinarm exoskeleton robot*	**Attention** *Behavioural Inattention Test (BIT)*	45 6:5–19:6 years 15 females CP type unk 45 unilateral MACS: 11 I, 18 II, 16 unk	**+ (significative)** Hawe et al. (2020) [47]—LOE 4
	**d440 Fine hand use** *Computerised Peg Moving Task (CPMT)*	**Visual perception** *Neuropsychological battery*	30 7:0–8:0 years 12 females 29 spastic, 1 mixed 20 unilateral, 10 bilateral Motor ability unk	**+ (significative)** Dellatolas et al. (2005) [44]—LOE 4

Abbreviations: − (significative), negative tendency with significative results; + (significative), positive tendency with significative results; ? (significative), tendency no specified; CP, cerebral palsy; GMFCS, Gross Motor Function Classification System; ICF, International Classification of Functioning, Disability and Health; LOE, level of evidence; MACS, Manual Ability Classification System; n.s., no significative results; unk, unknown; italic format, measures used; *, detailed results are presented in Supplementary Table S3.

#### 3.3.5. Self-Care (d5)

This ICF chapter addresses an individual’s capacity to manage daily self-care activities, including personal hygiene, dressing, eating, drinking, toileting, and maintaining health and safety. Two studies rated at OCEBM level 4 and one at level 3 examined the association between Self-care (d5) and general intellectual functioning in children and adolescents from about 5 to 18 years of age with predominantly spastic CP (*n* = 300) (Table 6).

Two of these studies used the RCPM as a measure of general intellectual functioning and the Pediatric Evaluation of Disability Inventory (PEDI) to assess self-care abilities [42,43]. Smits et al. [42] found that cognitive function was a significant predictor of personal care performance, even after accounting for gross motor function. In this prospective longitudinal cohort, children with higher intellectual capacity demonstrated the greatest gains in self-care over time, a finding consistent with the positive correlation between RCPM and PEDI scores reported by Burgess et al. [43]. Additionally, Burgess et al. [43] noted that both fine hand mobility and cognitive ability approached significance in predicting self-care performance in multiple regression analyses. These results also align with Milićević [48], who described significant correlations between lower intellectual functioning and reduced involvement in personal care activities. However, no significant relationship was found between general intellectual functioning and the frequency (ranging from never to daily) of self-care participation. In general, the studies included individuals with dyskinetic CP (*n* = 30) and encompassed all GMFCS levels, which positions self-care as a well-studied chapter of the ICF in representative samples of the CP population regarding CP type and motor severity.

Overall, the evidence suggests that general intellectual functioning plays a role in children and adolescents’ ability to perform self-care tasks, but not necessarily in how often they participate in these activities. This relationship supports the integration of cognitive assessment into programs aimed at fostering personal autonomy, allowing for the adjustment of support intensity or instruction based on each child’s cognitive profile.

**Table 6:** Self-Care (d5).

ICF		Cognitive Assessment	Demographic Data	Main Results *
**ICF chapter**	**ICF second level** *Assessment*	**Cognitive domain** *Instrument*	n Age range (years:months) n females n type CP n pattern CP Motor ability	**+ (significative)** **− (significative)** **n.s.** Author (year)—LOE
**d5 Self-care**	**d510–d599 Self-care** *Participation and Environment Measure for Children and Youth (PEM-CY)* *Pediatric Evaluation of Disability Inventory–Computer Adaptive Test, Speedy Version (PEDI-CAT)* **d510 Washing oneself** **d520 Caring for body parts** **d530 Toileting** **d540 Dressing** **d550 Eating** **d560 Drinking** *Pediatric Evaluation of Disability Inventory (PEDI)*	**General intellectual functioning** *Raven Coloured Progressive Matrices (RCPM)* *Revised Scale for Measuring Intelligence according to Wechsler principles (REVISK)*	300 7:0–18:0 years 115 females 242 spastic, 30 dyskinetic, 18 ataxic, 10 mixed 87 unilateral, 155 bilateral, 58 unk GMFCS: 115 I, 67 II, 47 III, 42 IV, 29 V MACS: 75 I, 103 II, 49 III, 36 IV, 27 V, 10 unk	**+ (significative)** Burgess et al. (2021) [43]—LOE 4 Milićević (2020) [48]—LOE 4 Smits et al. (2011) [42]—LOE 3 **n.s.** Milićević (2020) [48]—LOE 4

Abbreviations: − (significative), negative tendency with significative results; + (significative), positive tendency with significative results; CP, cerebral palsy; GMFCS, Gross Motor Function Classification System; ICF, International Classification of Functioning, Disability and Health; LOE, level of evidence; MACS, Manual Ability Classification System; n.s., no significative results; unk, unknown; italic format, measures used; *, detailed results are presented in Supplementary Table S3.

#### 3.3.6. Interpersonal Interactions and Relationships (d7)

This chapter explores the skills needed to initiate, maintain, and manage relationships, from basic social interactions to close family bonds and intimate partnerships, reflecting the social dimension of functioning. The association between Interpersonal interactions (d7) and cognition was studied by four articles: three addressing general intellectual functioning and one executive function (Table 7). One study was rated OCEBM LOE 3, with the others at level 4.

Among 157 children around 5 to 12 years, higher general intellectual functioning was significantly associated with greater social adjustment and functionality [42,49]. However, no significant associations were found between cognitive ability—measured by the vocabulary subtest of the Wechsler scales—and aspects such as emotional support or peer problems in friendships with outcomes derived from self-assessments [49]. In adolescents (*n* = 160, mean age 15.4 years), higher Intelligence Quotient (IQ) was linked to better prosocial behavior and fewer hyperactivity symptoms, as measured by the Strengths and Difficulties Questionnaire (SDQ), though no significant correlations were found with emotional symptoms or peer and conduct problems [50]. All GMFCS levels were represented, and two studies included individuals with dyskinetic CP (*n* = 20).

Regarding executive functions in 46 children and adolescents between 8 and 16 years with mild (GMFCS I–II) unilateral CP, decreased executive abilities were associated with increased risk of prosocial difficulties, hyperactivity symptoms, and even behavioral disorders, but not with emotional difficulties or peer problems [28].

Overall, these findings indicate that higher cognitive ability is linked to better performance in certain aspects of interpersonal and socioemotional functioning. However, other dimensions related to emotional aspects and peer problems—whether reported by parents or self-reports—are not equally influenced. These findings highlight that some aspects of interpersonal functioning may require specific cognitive interventions.

**Table 7:** Interpersonal Interactions and Relationships (d7).

ICF		Cognitive Assessment	Demographic Data	Main Results *
**ICF chapter**	**ICF second level** *Assessment*	**Cognitive domain** *Instrument*	n Age range (years:months) n females n type CP n pattern CP Motor ability	**+ (significative)** **− (significative)** **n.s.** Author (year)—LOE
**d7 Interpersonal interactions and relationships**	**d710–d729 General interpersonal interactions** *Pediatric Evaluation of Disability Inventory (PEDI)* **d710 Basic interpersonal interactions** *Friendship Quality Questionnaire (FQQ)* *Personality Inventory for Children—2nd edition (PIC-2)* **d720 Complex interpersonal interactions** *Strengths and Difficulties Questionnaire (SDQ)*	**General intellectual functioning** *Leiter International Performance Scale—Revised (Leiter-R)* *Raven Coloured Progressive Matrices (RCPM)* *Wechsler Intelligence Scale for Children—3rd edition (WISC-III)*	317 4:8–19:0 years 123 females 255 spastic, 20 dyskinetic, 4 ataxic, 38 unk 94 unilateral, 161 bilateral, 62 unk GMFCS: 61 I, 21 II, 34 III, 14 IV, 15 V, 111 I–III, 49 IV–V, 12 unk MACS: 80 I, 80 II, 14 III, 8 IV, 11 V, 124 unk	**+ (significative)** Brossard-Racine et al. (2013) [50]—LOE 4 Cunningham et al. (2009) [49]—LOE 4 Smits et al. (2011) [42]—LOE 3 **− (significative)** Brossard-Racine et al. (2013) [50]—LOE 4 **n.s.** Brossard-Racine et al. (2013) [50]—LOE 4 Cunningham et al. (2009) [49]—LOE 4
	**d720 Complex interpersonal interactions** *Strengths and Difficulties Questionnaire (SDQ)*	**Executive functions** *Delis–Kaplan Executive Function System (D-KEFS)* *Rey Complex Figure Test (RCFT)* *Test of Everyday Attention for Children (TEA-Ch)* *Wechsler Intelligence Scale for Children—4th edition (WISC-IV)*	46 8:0–16:0 years 21 females CP type unk 46 unilateral GMFCS: 35 I, 11 II MACS: 6 I, 40 II	**− (significative)** Whittingham et al. (2014) [28]—LOE 4 **n.s.** Whittingham et al. (2014) [28]—LOE 4

Abbreviations: − (significative), negative tendency with significative results; + (significative), positive tendency with significative results; CP, cerebral palsy; GMFCS, Gross Motor Function Classification System; ICF, International Classification of Functioning, Disability and Health; LOE, level of evidence; MACS, Manual Ability Classification System; n.s., no significative results; unk, unknown; italic format, measures used; *, detailed results are presented in Supplementary Table S3.

#### 3.3.7. Major Life Areas (d8)

This chapter encompasses participation in education, employment, and financial management, emphasizing the individual’s engagement in structured life roles and responsibilities essential for independence and social integration. Only one study in this review addressed the ICF domain of Major life areas (d8), with an OCEBM LOE 4 [51] (Table 8). In a sample of 148 children and adolescents (6.1–13.6 years range) with primarily spastic CP and GMFCS II–IV, significant positive correlations were found between specific cognitive domains, particularly memory and understanding, and participation in school settings. Children with better cognitive performance were more actively involved and independent in various school activities.

In summary, although the scarce current evidence suggests that certain cognitive skills, such as memory and comprehension, are positively associated with participation in educational contexts, more research is needed that focuses specifically on these crucial life areas to better understand and support the participation and independence of individuals with CP.

#### 3.3.8. Community, Social and Civic Life (d9)

This section focuses on involvement in community, leisure, religious, political, and cultural activities, reflecting the broader aspects of social life and active participation in society. Four articles examined the relationship between activity participation in Community, social and civic life (d9) and general intellectual functioning or cognitive development in 312 children and adolescents with an age range between 2 and 18 years, covering all GMFCS levels [48,52,53,54], with all but one study [54] (LOE 3) rated at OCEBM LOE 4 (Table 9). Cross-sectional studies reported higher IQ levels associated with participation in a greater and more diverse range of unplanned activities, as well as a preference for activities involving physical engagement. Interestingly, children with lower intellectual levels significantly report higher enjoyment of spontaneous activities with a tendency to recreational ones [52,53].

Referring to home-based activities, IQ was significantly related to both the frequency and degree of involvement in participation, with a stronger association for involvement [48]. Conversely, lower IQ levels were linked to reduced participation in social and home activities [48,52]. From a longitudinal perspective, cognitive ability was found to be a negative predictor of change in the diversity of social activity participation over time, potentially reflecting a ceiling effect; children with higher cognitive abilities may already participate at higher levels, leaving more room for improvement among those with cognitive impairments [54].

In summary, intellectual functioning appears to be potentially related to both the level and type of participation in leisure, home, and school activities, with differences depending on cognitive ability and the time frame considered. Knowledge of the cognitive profile in children and adolescents with CP can inform the development of adapted leisure and community participation activities, optimizing accessibility and opportunities for social inclusion outside the school environment.

**Table 9:** Community, Social and Civic Life (d9).

ICF		Cognitive Assessment	Demographic Data	Main Results *
**ICF chapter**	**ICF second level** *Assessment*	**Cognitive domain** *Instrument*	n Age range (years:months) n females n type CP n pattern CP Motor ability	**+ (significative)** **− (significative)** **n.s.** Author (year)—LOE
**d9 Community, social and civic life**	**d920 Recreation and leisure** *Assessment of Preschool Children’s Participation (APCP)* *Children’s Assessment of Participation and Enjoyment (CAPE)* *Participation and Environment Measure for Children and Youth (PEM-CY)* *Preferences for Activities of Children (PAC)*	**General intellectual functioning** *Leiter International Performance Scale—Revised (Leiter-R)* *Revised Scale for Measuring Intelligence according to Wechsler principles (REVISK)* **Cognitive development** *Comprehensive Developmental Inventory for Infants and Toddlers (CDIIT)*	312 2:0–18:0 years 123 females 181 spastic, 12 dyskinetic, 11 ataxic, 10 mixed, 11 other, 87 unk 71 unilateral, 190 bilateral, 11 other, 40 unk GMFCS: 112 I, 72 II, 34 III, 41 IV, 36 V, 5 III–IV, 12 III–V MACS: 12 I, 35 II, 19 III, 28 IV, 16 V, 202 unk	**+ (significative)** Majnemer et al. (2008) [52]—LOE 4 Majnemer et al. (2010) [53]—LOE 4 Milićević (2020) [48]—LOE 4 **− (significative)** Majnemer et al. (2008) [52]—LOE 4 Milićević (2020) [48]—LOE 4 Wu et al. (2015) [54]—LOE 3

Abbreviations: − (significative), negative tendency with significative results; + (significative), positive tendency with significative results; CP, cerebral palsy; GMFCS, Gross Motor Function Classification System; ICF, International Classification of Functioning, Disability and Health; LOE, level of evidence; MACS, Manual Ability Classification System; n.s., no significative results; unk, unknown; italic format, measures used; *, detailed results are presented in Supplementary Table S3.

### 3.4. Environmental Factors

#### 3.4.1. Products and Technology (e1)

This chapter includes the physical tools, devices, equipment, and technologies—ranging from assistive aids to everyday appliances—that influence an individual’s functioning either as facilitators or barriers. In the context of children with CP, the study by Pirila et al. [46] with an OCEBM LOE of 4, underscores the relevance of communication-related Products and technology (e1) in supporting cognitive development and performance in a sample of 36 children with a mean age of 5 years and 1 month, including all levels of GMFCS (Table 10).

The findings revealed a positive association between the use of augmentative and alternative communication (AAC) aids and cognitive abilities, suggesting that access to appropriate technological supports may enable children to more effectively express their cognitive capacities—such as attention, memory, and problem-solving—or even contribute to their improvement. These results highlight the importance of selecting and adapting assistive products and technologies based on a child’s cognitive profile to optimize functional independence and participation. Early integration of cognitive, including language, assessments can guide clinicians in recommending appropriate technologies, such as communication devices or mobility aids.

**Table 10:** Environmental Factors (e).

ICF		Cognitive Assessment	Demographic Data	Main Results *
**ICF chapter**	**ICF second level** *Assessment*	**Cognitive domain** *Instrument*	n Age range (years:months) n females n type CP n pattern CP Motor ability	**+ (significative)** **− (significative)** **n.s.** Author (year)—LOE
**e1 Products and technology**	**e125 Products and technology for communication** *Augmentative and Alternative Communication* *(AAC)*	**General intellectual functioning** *Griffiths Scales of Mental Development (GSMD)* *Wechsler Intelligence Scale for Children—Revised (WISC-R)* *Wechsler Preschool and Primary Scales of Intelligence—Revised (WPPSI-R)*	36 1:10–9:0 years 16 females 22 spastic, 14 unk 5 unilateral, 31 bilateral GMFCS: 7 III, 12 I–II, 17 IV–V	**+ (significative)** Pirila et al. (2007) [46]—LOE 4
**e3 Support and relationships**	**e320 Friends** *Social Network Inventory for Children (SNIC)*	**General intellectual functioning** *Wechsler Intelligence Scale for Children—3rd edition (WISC-III)*	41 6:0–12:0 years 18 females 38 spastic, 3 unk 10 unilateral, 28 bilateral, 3 unk GMFCS: 5 I, 1 II, 17 III, 5 IV, 1 V, 12 unk	**+ (significative)** Cunningham et al. (2009) [49]—LOE 4
**e4 Attitudes**	**e410 Individual attitudes of immediate family members** *Family Empowerment Scale (FES)* *Parenting Dimensions Inventory (PDI)*	**General intellectual functioning** *Wechsler Intelligence Scale for Children—3rd edition (WISC-III)* **Cognitive development** *Bayley Scales of Infant and Toddler Development—3rd edition (BSID-III)*	82 1:1–12:0 years 36 females 38 spastic, 44 unk 10 unilateral, 28 bilateral, 44 unk GMFCS: 9 I, 20 II, 26 III, 14 IV, 1 V, 12 unk	**+ (significative)** Pierce et al. (2023) [55]—LOE 4 **n.s.** Cunningham et al. (2009) [49]—LOE 4
	**e430 Individual attitudes of people in positions of authority** *Teacher reading expectations* *Teacher writing expectations*	**General intellectual functioning** *Raven Coloured Progressive Matrices (RCPM)*	49 71.88 ± 5.82 months ^‡^ 18 females 48 spastic, 1 ataxic 7 unilateral, 40 bilateral, 2 unk Motor ability unk	**+ (significative)** Peeters et al. (2009) [41]—LOE 4
	**e430 Individual attitudes of people in positions of authority** *Teacher reading expectations* *Teacher writing expectations*	**Language** *Dutch Language Proficiency Test* *Peabody Picture Vocabulary Test—3rd edition (PPVT-III)*	49 71.88 ± 5.82 months ^‡^ 18 females 48 spastic, 1 ataxic 7 unilateral, 40 bilateral, 2 unk Motor ability unk	**+ (significative)** Peeters et al. (2009) [41]—LOE 4 **n.s.** Peeters et al. (2009) [41]—LOE 4
**e5 Services, systems, and policies**	**e580 Health services, systems, and policies** *Formal questionnaire regarding current educational and rehabilitation services* *Speech therapy*	**General intellectual functioning** *Griffiths Scales of Mental Development (GSMD)* *Leiter International Performance Scale—Revised (Leiter-R)* *Wechsler Intelligence Scale for Children—Revised (WISC-R)* *Wechsler Preschool and Primary Scales of Intelligence—Revised (WPPSI-R)*	294 1:10–19:0 years 118 females 22 spastic, 272 unk 5 unilateral, 31 bilateral, 258 unk GMFCS: 94 I, 7 III, 8 IV, 24 V, 12 I–II, 71 II–III, 70 IV-V, 8 unk	**− (significative)** Pirila et al. (2007) [46]—LOE 4 Majnemer et al. (2014) [56]—LOE 4

Abbreviations: − (significative), negative tendency with significative results; + (significative), positive tendency with significative results; CP, cerebral palsy; GMFCS, Gross Motor Function Classification System; ICF, International Classification of Functioning, Disability and Health; LOE, level of evidence; n.s., no significative results; unk, unknown; italic format, measures used; ^‡^, mean ± standard deviation; *, detailed results are presented in Supplementary Table S3.

#### 3.4.2. Support and Relationships (e3)

The Support and relationships (e3) chapter looks at the role of family, friends, caregivers, peers, and others who provide emotional, physical, or social support, contributing significantly to a person’s ability to function and participate. In relation to this environmental factor, with an OCEBM LOE of 4, the study by Cunningham et al. [49], with a sample of 41 children (mean age 8.76 years), primarily diagnosed with spastic CP and classified at GMFCS level III, although all levels were represented, also examined children’s social networks, specifically regarding friendship (Table 10), and found a positive significant correlation with cognitive ability.

This suggests that better cognitive performance may be associated with having more friends and greater interaction with them, which reinforces the potential for cognitive-focused interventions to transfer benefits to other areas of daily life.

#### 3.4.3. Attitudes (e4)

This ICF chapter addresses the beliefs, perceptions, and values held by individuals, communities, and institutions that can either enable or hinder the participation and inclusion of people with diverse functioning. The relationship between individual attitudes (e4) and aspects of cognition has been explored in three articles included in this review (OCEBM LOE of 4) (Table 10). Within the family context, studies by Cunningham et al. [49] and by Pierce et al. [55], each involving samples of 41 participants, report contrasting findings. Cunningham et al. [49], with a sample of children with 8.76 years mean age and predominantly classified as GMFCS III, found that individual attitudes of immediate family members—reflected in parenting behaviors and attitudes—were not significantly correlated with children’s cognitive ability as measured by the vocabulary subtest of the Wechsler scales. In contrast, Pierce et al. [55] in their sample with a mean age of 23.8 m and largely classified as GMFCS level II, observed that greater cognitive development in children was positively associated with higher levels of perceived family empowerment, suggesting a link between a child’s cognitive functioning and their family’s sense of competence in managing life situations.

Attitudes within the school environment were examined by Peeters et al. [41] in a sample of 49 children (mean age = 71.88 months). Their findings indicate that teachers’ expectations regarding students’ reading and writing abilities—representing the attitudes of individuals in positions of authority, according to the ICF terms—were positively correlated with students’ intellectual functioning. Notably, only reading expectations showed a significant positive correlation with language-related cognitive domains (vocabulary and syntactic skills). Intelligence emerged as the primary or sole predictor of teachers’ reading and writing expectations in the tested models.

Taken together, these studies suggest that while parenting style or attitudes alone may not be directly associated with cognitive outcomes, a higher intellectual function in children may be linked to greater family empowerment and more favorable academic expectations from teachers. These findings highlight the importance of supporting family empowerment and educating caregivers and teachers in understanding the child’s cognitive strengths and challenges to set realistic and positive expectations.

#### 3.4.4. Services, Systems, and Policies (e5)

This chapter covers broader societal structures, including health, education, labor, social welfare, and transportation systems, and the policies and regulations that govern them, shaping opportunities and constraints in daily life. Within this domain, two studies, including individuals across all GMFCS levels, with an OCEBM LOE of 4, explored how access to and use of services (e5) are influenced by cognitive functioning (Table 10). Majnemer et al. [56], in a sample of school-age children (*n* = 91, age range of 6 to 12 years), found that those with lower IQ scores tended to receive a higher number of therapeutic services, including speech–language pathology, occupational therapy, and physical therapy. Similarly, Pirila et al. [46], working with a younger sample of 36 children aged 1 to 9 years, also reported that children with lower levels of intelligence received more speech therapy interventions and made greater use of (AAC) systems. In adolescents (*n* = 167, age range of 12 to 19 years), this pattern extended to services related to psychology and special education.

Overall, lower performance in general intellectual functioning appears to be associated with an increased reception of therapeutic services and communication support systems, reflecting the role of public services and policies in meeting the needs of individuals with lower cognitive performance. These findings underscore the importance of tailored service provision within health, education, and social welfare systems that align with cognitive functioning levels. Therefore, early cognitive assessment should guide individualized care plans to optimize resource allocation and ensure timely, comprehensive support.

Within the broader component of Services, systems, and policies (e5), particular attention has been given to health-related services and policies, which play a central role in shaping access to care and rehabilitation for individuals with diverse cognitive profiles. The specific second-level category e580. Health services, systems, and policies refer to the organization, availability, and regulation of healthcare services that directly impact medical care, rehabilitation, and overall support for functioning and development. The following section provides an in-depth examination of the available evidence on this topic, allowing for a more comprehensive exploration of the range of interventions that may be related not only to overall cognitive performance but also to specific cognitive functions.

##### Health Services, Systems, and Policies (e580)

To deepen understanding of this area, nine studies were identified that examined the effects of health interventions on cognition in children with CP. These studies used a wide variety of assessment tools—none repeated across articles—and addressed both general and specific cognitive functions. Eight studies were rated as OCEBM LOE 2 and one as LOE 4 (Table 11).

Five of the articles explored the effects of physical interventions, with durations ranging from one to 72 sessions. The cognitive domains most frequently assessed were attention or processing speed and executive functions [57,58,59,60], followed by memory [57] and cognitive development [24]. Except for the study on cognitive development—which focused on adolescents (*n* = 13; mean age = 17.07 y; GMFCS levels II–V)—the rest primarily involved younger children with spastic CP (mean age ≈ 9 years; GMFCS levels I–III). Results in attention and processing speed were generally positive, with improvements mainly in reaction time and perseveration [57,58,60], suggesting that exercise and yoga-based interventions may support attentional functioning.

However, findings related to executive functions were mixed: while AL-Nemr [57] reported significant improvements in reaction behavior and logical reasoning following strength training combined with physical therapy, Maltais et al. [60] found that intense exercise worsened inhibitory control. Other executive function measures (inhibition, response accuracy, switching) showed no significant changes [58,60]. Memory outcomes also followed a trend of improvement consistent with attention and processing speed findings [57], and cognitive development appeared to benefit significantly from dance-based intervention [24]. These results suggest that attention, processing speed, memory, and cognitive development may be positively influenced by physical interventions, although the impact on executive functions remains ambiguous. Regarding the maintenance of these effects, follow-up assessments by AL-Nemr [57] and Mak et al. [59] found no significant long-term gains, suggesting that the improvements observed immediately post-intervention did not persist six months later.

Three studies evaluated the impact of biofeedback and brain stimulation interventions. These included 10 to 36 sessions targeting visual perception [61,62], attention [62], and general intellectual functioning [63], in children aged between 6.43 and 10.2 years, mostly with spastic CP and GMFCS levels I–III when reported. Alwhaibi et al. [61] observed significant improvements in visual perception following a combined physical therapy and biofeedback approach. Chen et al. [62] found selective effects using neurofeedback, with improvements in visual sequential memory and visual closure, as well as reduced omission errors in sustained attention, although no other attentional components improved. Regarding general intellectual functioning, Collange-Grecco et al. [63] used transcranial direct current stimulation (tDCS) combined with other therapies and found no immediate post-intervention effects but observed delayed cognitive gains one month later. These results suggest that specific components of visual perception and attention may be enhanced through biofeedback, while tDCS may support intellectual functioning over time.

Lastly, Hardy et al. [64] assessed the effects of a medical intervention—40 sessions of hyperbaric oxygen therapy—on attention and executive functioning in 40 children with spastic CP (ages 4–12). While overall attentional improvements were limited, a significant enhancement was noted in auditory correct responses, both immediately post-intervention and at the three-month follow-up. Executive functioning showed broader improvements (reaction behavior, logical reasoning, self-control, and visual span) that were occasionally maintained over a three-month follow-up.

In addition to interventions targeting cognition through physical, medical, and neuromodulatory interventions, another line of research explored how cognitive interventions may influence non-cognitive aspects of functioning. Within this framework, four studies examined the effects of cognitive interventions, as an Environmental Factor (e) on other non-cognitive ICF components, the Activities and Participation (d) in children with CP, including two classified as OCEBM LOE 2, one as LOE 3, and one as LOE 4 (Table 12). In all cases, the interventions were delivered via computerized programs [40,65,66,67].

Specifically, the studies that focused on the General tasks and demands (d2) domain explored the impact of these cognitive interventions on children’s ability to carry out daily routines [40,65,67], employing similar evaluation tools across studies, particularly the BRIEF questionnaire, which was used in all three. Among the 56 children included, predominantly with mild to moderate motor impairment, results indicated non-significant improvements in this domain following cognitive training programs lasting between 2 and 12 weeks.

Further assessments were conducted in other domains such as Interpersonal interactions and relationships (d7) and Communication (d3). Wotherspoon et al. [67] evaluated the effects of the intervention on social behavior and the use of communication devices and techniques, again finding no significant post-intervention improvements. Similarly, participation in Recreation and leisure activities (d9) was assessed by Blasco et al. [66] in a sample of 30 children (mean age: 10 years and 4 months), with no significant changes observed either immediately after the 12-week intervention or at a 9-month follow-up.

The results consistently showed no significant improvements in the competencies of handling daily routines, relational behavior, and communication following the different cognitive training programs.

**Table 11:** Health Services, Systems, and Policies (e580): Health interventions on cognition.

Intervention		Cognitive Assessment	Demographic Data	Main Results *
**Type**	*Intervention’s name* Characteristics	**Cognitive domain** *Instrument*	n (IG/CG) Age range (years:months) n females (IG/CG) n type CP n pattern CP Motor ability	**+ (significative)** **− (significative)** **n.s.** Author (year)—LOE
**Physical**	*Intense exercise* Intense aerobic exercise that consists of a shuttle run and walk test 1 session	**Executive functions/Processing speed** *Stroop-like test, modified and designed for children*	17 (8/9) 6:0–14:8 years 7 females (3/4) 8 spastic CP pattern unk GMFCS: 8 I	**+ (significative)** Maltais et al. (2016) [60]—LOE 4 **− (significative)** Maltais et al. (2016) [60]—LOE 4 **n.s.** Maltais et al. (2016) [60]—LOE 4
	*Dance intervention* Physical intervention (coordination movements of upper and lower limbs, body image interaction between subject and environment, skill and agility sequential components of the movement, and trunk and head movements for spatial orientation and equilibrium) 2 sessions of 60 min, twice per week, for 3 months	**Cognitive development** *Functional Independence Measure (FIM)*	26 (13/13) 15:0–29:0 years 15 females (8/7) CP type unk CP pattern unk GMFCS: 3 II, 5 III, 4 IV, 1 V	**+ (significative)** Teixeira-Machado et al. (2017) [24]—LOE 2
	*MiYoga* Mindfulness and mindful movement techniques based on hatha yoga principles 6 sessions of 90 min, for 6 weeks, with 2 follow-up consultations via phone or Skype over the following 2 weeks	**Attention** *Conners’ Continuous Performance Test—2nd edition (CPT-II)* *Wechsler Intelligence Scale for Children—4th edition (WISC-IV)* **Executive functions** *Delis–Kaplan Executive Function System (D-KEFS)* *Wechsler Intelligence Scale for Children—4th edition (WISC-IV)*	42 (21/21) 6:0–16:0 years 18 females (7/11) 21 spastic 7 unilateral, 14 bilateral GMFCS: 11 I, 4 II, 6 III	**+ (significative)** Mak et al. (2018) [58]—LOE 2 **n.s.** Mak et al. (2018) [58]—LOE 2
	*MiYoga* Mindfulness and mindful movement techniques based on hatha yoga principles 6 sessions of 90 min, for 6 weeks, with 2 follow-up consultations via phone or Skype over the following 2 weeks	**Attention** *Conners’ Continuous Performance Test—2nd edition (CPT-II)* *Wechsler Intelligence Scale for Children—4th edition (WISC-IV)* **Executive functions** *Delis–Kaplan Executive Function System (D-KEFS)* *Wechsler Intelligence Scale for Children—4th edition (WISC-IV)*	23 6:0–16:0 years 0 females 23 spastic 10 unilateral, 13 bilateral GMFCS: 14 I, 6 II, 3 III	**n.s.** Mak et al. (2022)—[59] LOE 2
	*Functional Strength Training (FST)* Functional strength training for lower limbs followed by conventional physical therapy 3 sessions of 90 min each per week, for 6 months	**Attention/Processing speed/Memory/Executive functions** *Computer-based RehaCom software (version 5)*	32 (16/16) 8:0–12:0 years 14 females (9/5) 16 spastic 16 bilateral GMFCS: 6 II, 10 III	**+ (significative)** AL-Nemr (2024) [57]—LOE 2 **n.s.** AL-Nemr (2024) [57]—LOE 2
**Biofeedback and brain stimulation**	*Augmented biofeedback* E-Link Upper Limb Exerciser, a computerized graded interactive system *Physical training* Exercises facilitating hand–eye coordination and fine motor skills 1 session of 60 min per day, three times per week, for 3 months	**Visual perception** *Beery–Buktenica Developmental Test of Visual–Motor Integration—6th edition (Beery)*	45 (15/30) 5:5–7:9 years 22 females (5/17) 15 spastic 15 unilateral MACS: 15 I–II	**+ (significative)** Alwhaibi et al. (2020) [61]—LOE 2
	*Transcranial Direct Current Stimulation (tDCS)* tDCS combined with treadmill training and training in intellectual activities 10 sessions	**General intelligence functioning** *Raven’s Coloured Progressive Matrices (RCPM)*	30 (15/15) 6:0–12:0 years unk females 15 spastic 6 unilateral, 9 bilateral GMFCS: 4 I, 7 II, 4 III	**+ (significative)** Collange-Grecco et al. (2023) [63]—LOE 2 **n.s.** Collange-Grecco et al. (2023) [63]—LOE 2
	*EEG Neurofeedback training* Neurofeedback 2 sessions of approximately 1 h each, for 10 weeks	**Attention** *Conners Continuous Performance Test—2nd edition (CPT-II)* **Visual perception** *Test of Visual–Perceptual Skills—3rd edition (TVPS-3)*	19 (8/11) 4:0–12:0 years 2 females (1/1) CP type unk 4 unilateral, 4 bilateral GMFCS: 4 I, 1 II, 3 III MACS: 3 I, 5 II	**+ (significative)** Chen et al. (2024) [62]—LOE 2 **n.s.** Chen et al. (2024) [62]—LOE 2
**Medical**	*Hyperbaric oxygen treatment (HBO_2_)* IG: 100% oxygen at 1.75 atmospheres absolute (HBO_2_) CG: air (21% oxygen) at 1.3 atmospheres absolute (Sham) 40 sessions of 1 h of either HBO_2_ or Sham treatment, for 2 months	**Executive functions** *Corsi Blocks (CB)* *Picture Span Tests* *Test of Variables of Attention (TOVA), 10.8-min vigilant condition* *Word Span Test* **Attention** *Test of Variables of Attention (TOVA), 10.8-min vigilant condition*	75 (40/35) 4:0–12:0 years 41 females (21/20) 40 spastic 1 unilateral, 38 bilateral, 1 unk Motor ability unk	**+ (significative)** Hardy et al. (2002) [64]—LOE 2 **n.s.** Hardy et al. (2002) [64]—LOE 2

Abbreviations: − (significative), negative tendency with significative results; + (significative), positive tendency with significative results; CG, control group; CP; cerebral palsy; GMFCS, Gross Motor Function Classification System; IG, intervention group; LOE, level of evidence; MACS, Manual Ability Classification System; n.s., no significative results; unk, unknown; italic format, intervention’s name and measures used; *, detailed results are presented in Supplementary Table S4.

**Table 12:** Health Services, Systems, and Policies (e580): Cognitive interventions on non-cognitive aspects of functioning.

Intervention		ICF Assessment	Demographic Data	Main Results *
**Type**	*Intervention’s name* Characteristics	**ICF component** ICF chapter; ICF second level *Assessment*	n (IG/CG) Age range (years:months) n females (IG/CG) n type CP n pattern CP Motor ability	**+ (significative)** **− (significative)** **n.s.** Author (year)—LOE
**Cognitive**	*Guttman NeuroPersonalTrainer, Child Version* Online and individual intervention, adapted depending on the cognitive function baseline level 16 sessions of 1 h, 2 days a week, for 8 weeks	**d Activities and Participation** d2 General tasks and demands; d230 Carrying out daily routine *Behavior Rating Inventory of Executive Function (BRIEF)* *Conners rating scales (CPRS-48/CTRS-28)*	15 7:0–14:0 years 7 females 14 spastic, 1 ataxic 7 unilateral, 8 bilateral GMFCS: 6 I, 4 II, 2 III, 3 V	**n.s.** Muriel et al. (2014) [40]—LOE 4
	*CogMed RM computer program* Computerized cognitive training Around 25 sessions of 30–40 min, 5 days a week, for 5 weeks	**d Activities and Participation** d2 General tasks and demands; d230 Carrying out daily routine *ADHD rating scale IV* *Behavior Rating Inventory of Executive Function (BRIEF)*	66 (32/34) 11.4 ± 3.1/9.4 ± 2.6 years ^‡^ 25 females (13/12) CP type unk CP pattern unk Motor ability unk	**n.s.** Beneventi et al. (2023) [65]—LOE 3
	*Strengthening Mental Abilities Through Relational Training (SMART)* Online cognitive training program Participants could complete 5 modules per day, with a total of 55 modules to complete up to 12 weeks	**d Activities and Participation** d2 General tasks and demands; d230 Carrying out daily routine *Behavior Rating Inventory of Executive Function (BRIEF)* *Conners-3 Rating Scale* d3 Communication; d350–369 Conversation and use of communication devices and techniques *Social Communication Questionnaire–Current (SCQ–Current–Parent form)* d7 Interpersonal interactions and relationships; d720 Complex interpersonal interactions *Behavior Assessment System for Children—3rd edition (BASC-3)* *Strengths and Difficulties Questionnaire (SDQ)*	21 (9/12) 4 females (whole sample) 8:3–12:6 years CP type unk CP pattern unk Motor ability unk	**n.s.** Wotherspoon et al. (2024) [67]—LOE 2
	*Neuronup* Home-based computerized executive function intervention 10 sessions of 15 min per week, for 12 weeks	**d Activities and Participation** d9 Community, social and civic life; d920 Recreation and leisure *Participation and Environment Measure for Children and Youth Questionnaire (PEM-CY)*	60 (30/30) 8:11–12:11 years 30 females (15/15) 27 spastic, 3 dyskinetic 17 unilateral, 10 bilateral, 3 unk GMFCS: 20 I, 6 II, 4 III MACS: 11 I, 16 II, 3 III	**n.s.** Blasco et al. (2025) [66]—LOE 2

Abbreviations: − (significative), negative tendency with significative results; + (significative), positive tendency with significative results; ADHD, Attention-Deficit/Hyperactivity Disorder; CG, control group; CP; cerebral palsy; CPRS-48, Conners Parent Rating Scale-48 items; CTRS-28, Conners Teacher Rating Scale-28 items; GMFCS, Gross Motor Function Classification System; ICF, International Classification of Functioning, Disability and Health; IG, intervention group; LOE, level of evidence; MACS, Manual Ability Classification System; n.s., no significative results; unk, unknown; italic format, intervention’s name and measures used; ^‡^, mean ± standard deviation; *, detailed results are presented in Supplementary Table S5.

Taken together, these studies suggest that although healthcare systems are increasingly incorporating cognitive interventions as part of rehabilitation services for children with CP, their isolated use may have limited impact on broader aspects of functioning related to daily routines, communication, social interaction and participation, highlighting the importance of evaluating both service provision and therapeutic effectiveness within the ICF framework.

These findings highlight the critical role of health services, systems, and policies in shaping cognitive and functional outcomes in people with CP. Therefore, comprehensive rehabilitation plans should integrate multidisciplinary approaches that combine physical, cognitive, and psychosocial interventions, tailored to individual cognitive profiles.

## 4. Discussion

This systematic review aimed to examine the contribution of various cognitive domains to the ICF components of Activities and Participation and Environmental Factors in people with CP. The analysis of the 44 studies included provides consistent evidence suggesting that cognitive functioning is meaningfully associated with multiple aspects of daily functioning in CP.

The most robust and recurrent associations emerged between cognitive abilities—particularly general intellectual functioning, language, and visual perception—and the ICF chapters of Mobility, Communication, and Learning and applying knowledge. In line with existing literature [3,6], this review reinforces the importance of cognitive functions in understanding and supporting autonomy and social participation in CP. More specifically, manual ability in individuals with bilateral CP showed the clearest associations with cognitive performance (general cognitive functioning, language, and visual perception), supporting previous findings that highlight the interconnectedness of motor and cognitive development. Communication outcomes were consistently linked to intellectual functioning and language skills across diverse CP profiles, and emergent literacy was notably associated with both home and school settings in children with primarily spastic CP, pointing to the relevance of these contexts in cognitive development.

The Environmental Factors component, while underrepresented compared to Activities and Participation, revealed meaningful links with support systems, social attitudes, and service provision, particularly in relation to general cognitive ability and language. This aligns with evidence suggesting that contextual factors such as access to assistive technology, school expectations, and family empowerment can act as facilitators or barriers to functioning [11,13].

In the present review, the ICF component of Activities and Participation was addressed in a larger number of studies compared to the Environmental Factors component. In the context of CP, this finding is consistent with results reported by Santana et al. [12], whose scoping review, which focused on adolescents and young adults (ages 13–30), identified a greater emphasis in the literature on domains related to health development, social participation, functional autonomy, and independent living. This trend also aligns with the composition of the ICF Core Sets for children and youth with CP [11], as well as the preliminary selection process for the adult CP Core Sets [68], both of which predominantly include categories from the Activities and Participation component.

Delving deeper, the Mobility chapter (d4) has been the most frequently studied, particularly in terms of “Changing and maintaining body position” and “Walking and moving” categories. More specifically, this ICF chapter has been extensively analyzed within the age range of 0–12 years, which not only makes sense considering that the diagnosis of CP is generally made within the early years of this range [69], and that mobility-related aspects are key to this [70], but also because age is a demographic for which mobility is a central developmental concern [68]. As noted by Santana et al. [12], this overrepresentation of the Activities and Participation component—especially motor aspects—may partly stem from the widespread use of functional classification tools such as the GMFCS, MACS, and CFCS, which are inherently aligned with this ICF component.

On the other hand, although the Environmental Factors component has been less represented overall, the category of Health services, systems, and policies (e5) was more frequently addressed, particularly in studies of health and cognitive interventions. Notably, while cognitive interventions did not consistently yield significant improvements in daily functioning, their inclusion reflects a growing interest in enhancing autonomy and personal development—moving beyond purely functional targets. This is not only consistent with the priorities expressed by individuals with CP themselves, as described by Palisano et al. [71], who emphasized the value of interventions that promote capability and participation across life domains. But also with the frequency with which chapter e5 of the ICF is studied in individuals aged 7 to 18, which aligns with the fact that in CP, the requirement for complex care continues unabated from adolescence onward, and individuals cannot be fully disconnected from health care systems [72].

The results within the environmental domain have primarily focused on analyzing the impact of health interventions. However, the potential effects of other environmental facilitators and barriers on cognitive functioning have not been systematically examined. Several studies have identified various environmental factors, such as family ecology, financial support, school type, and parental stress, as being associated with participation levels in children and youth with CP, underscoring their relevance as modifiable determinants of functional outcomes [73,74]. Given their modifiable nature, interventions targeting environmental facilitators and barriers may represent a feasible and potentially fruitful approach to enhance cognitive and participation outcomes. Moreover, prioritizing these environmental factors for intervention and implementation is crucial to improving quality of life, as supported by research emphasizing the need for systematic assessment and targeted actions within clinical and social frameworks [75]. Nevertheless, empirical evidence on the direct influence of these environmental conditions on cognitive development and/or functioning remains limited. The current underrepresentation of environmental factors in CP research likely reflects an imbalance in the application of the ICF model, where this domain has received comparatively less attention despite its recognized relevance. Therefore, further investigation is needed to explore how modifiable environmental factors interact with cognitive functioning, with the aim of identifying the most relevant variables that should be prioritized for intervention and/or implementation.

Taking a global view of the ICF-related findings in this review, one key methodological aspect involves the tools used to assess the ICF components. In some studies, assessments were administered directly to the child or adolescent, while in others, data were collected via proxy reports. This distinction is particularly relevant in CP populations, where not all individuals are able to self-report due to motor, communicative, or cognitive limitations that may affect their ability to express personal experiences [76]. Notably, discrepancies between self- and proxy-reports have been documented in both neurotypical and CP samples, with caregivers typically providing less favorable evaluations of the child’s functioning [77,78]. These findings underscore the need for developing assessment tools that move beyond proxy-only formats and incorporate the individual’s perspective whenever possible, even in complex clinical populations [12].

Continuing with the analysis of the assessment tools, the focus now turns to the differences in how the components of the Activities and Participation and the Environmental Factors have been assessed: studies addressing the Activities and Participation component generally used standardized, ICF-aligned measures, while studies focusing on Environmental Factors were included even when they used non-standardized instruments. This more flexible inclusion criterion proved essential. Without it, valuable studies exploring real-world contextual influences—such as school type [56], therapy provision [45,56], teacher expectations [41], or use of augmentative communication systems [45]—would have been excluded. At the same time, this highlights a pressing need: the development and validation of standardized, ICF-compatible instruments to assess Environmental Factors, which would facilitate consistency and comparability across studies and contribute to building a more robust body of evidence in this underexplored domain.

Turning to cognition, the review considered a wide range of domains, including cognitive development, general intellectual functioning, language, executive functions, attention, visual perception, memory, processing speed, and social cognition. This aligns with known cognitive profiles in CP, where approximately half of individuals score below the normative range in IQ [4], and where deficits in visual perception, language, memory, and executive functioning are especially prevalent [6,21]. In keeping with Stadskleiv’s [3] observations, general intellectual functioning and language emerged as the most frequently assessed domains, while memory remained comparatively less studied—an imbalance also evident in the present review.

Regarding assessment tools, commonly used instruments for general cognition include the RCPM and Wechsler scales, both widely recognized in the field [6]. However, limitations have been noted when applying these tools in CP populations. Fluss and Lidzba [6] emphasize that visual perception and motor difficulties can compromise test validity, particularly in children with more severe impairments. In this context, nonverbal assessments like the RCPM are considered preferable for evaluating general cognitive performance, especially in cases of speech or motor limitations [79]. This is supported by studies included in this review (e.g., [33,42,43]), which demonstrate the RCPM’s sensitivity to relevant cognitive–functional associations. By contrast, instruments such as the Brief IQ Screener from the Leiter-R, as used by Koopmans et al. [32], were limited to children with CFCS levels I–III, excluding individuals with greater communicative impairment and thereby restricting the scope of analysis.

More broadly, there was marked heterogeneity in the tools used to assess specific cognitive domains. This variation reflects both the absence of universally accepted protocols for cognitive assessment in CP and the practical challenges posed by participant heterogeneity. Sand et al. [80] similarly highlighted this issue in their scoping review on cognitive assessment in adults with CP, advocating for the adoption of standardized neuropsychological batteries. Promising initiatives in this direction are already underway in Scandinavian countries, where Bottcher et al. [81] and Stadskleiv et al. [82] have described structured assessment protocols adapted for children and adults with CP.

Yet even when multiple instruments are available, assessments are not always feasible for all individuals. For example, Koopmans et al. [32] attempted to assess receptive language across a diverse sample using three instruments, but still encountered participants for whom no suitable measure could be applied. This study illustrates a critical need for accessible and high-quality standardized assessment tools that accommodate diverse communication modes and support inclusive cognitive profiling.

In addition to heterogeneity in tools, the review revealed significant variability in sample characteristics, including age, severity of CP, and presence of comorbidities. This complexity complicates the interpretation of the associations between cognition and ICF components. Consistent with Chagas et al. [83], only one study in this review included participants over 20 years of age. Most samples combined children and adolescents without stratifying by developmental stage, which limits the generalizability of findings to adults and runs counter to recommendations for age-specific analysis considering Brief ICF Core Sets specific to age groups [11].

Furthermore, many studies analyzed associations only within relatively homogeneous samples, often excluding individuals with moderate to severe intellectual or functional impairments. For example, in studies such as Peeters et al. [25,26,27] and Whittingham et al. [28], the associations found were based on samples with normative or near-normative performance profiles, suggesting that certain findings may not apply across the full spectrum of CP. Likewise, differences in outcomes were observed when the same language assessment was applied to samples with differing motor and speech severity levels, as seen in the contrasting results of Koopmans et al. [32] and Nordberg et al. [34]. These inconsistencies underline the importance of considering CP heterogeneity—including comorbidities—when interpreting data and highlight the value of analytical strategies that control for relevant covariates (e.g., [31]).

Despite the heterogeneity of the samples described above, throughout this systematic review, we have attempted to analyze results in relation to characteristics such as motor severity or the type of CP. Given that GMFCS levels IV–V and dyskinetic CP are often underrepresented in studies, an effort was made to identify patterns related to these variables. The ICF chapters in which more studies included samples with these characteristics or where these variables were more traceable were mainly Communication (d3), Mobility (d4), and Self-care (d5), with an emphasis on the fact that, in dyskinetic CP or when the condition becomes more severe, these ICF chapters appear to be more related to general cognitive functioning and language abilities. These findings are not surprising considering that dyskinetic CP is one of the most severe and disabling subtypes of the condition [84]. This type of CP is mainly characterized by the presence of dystonia and other associated impairments that can affect daily activities and participation [85,86]. On the other hand, the prevalence of impairments, such as intellectual, speech, hearing, and visual impairments, increases with GFMCS level [87]. Overall, these motor limitations may restrict individuals with CP from participating in daily activities, as those with this severe profile often have limited functional abilities and need to rely on others for assistance [88], which may contribute to explaining the relationships found in this review. Further studies are needed to investigate which specific cognitive functions may be related to functioning, particularly as a function of the severity or type of cerebral palsy, in order to inform and optimize intervention strategies.

Throughout the execution of this systematic review, careful attention was given to both methodological rigor and content quality. Firstly, a key strength lies in the definition of the inclusion time frame: the lower limit was set at 2002, corresponding to the publication of the current ICF version by the WHO, and the upper limit extended to 2025, thereby incorporating the most recent literature available. This broad time frame enabled the inclusion of a wide variety of studies—44 in total—allowing for a balanced representation of both foundational and more recent research. Indeed, over half of the included articles (52%) were published from 2015 onwards. In terms of sample characteristics, the participants analyzed across studies appear to be representative of the CP population in relation to sex [89,90], CP type [89], and motor severity [91]. Specifically, the aggregated sample was composed predominantly of males (58.8%), individuals with spastic CP (82.5%), and those with mild to moderate motor impairment (GMFCS levels I–III).

With regard to the content focus, and as is typical for systematic reviews, this article reflects trends in the existing literature—most notably, a stronger emphasis on the Activities and Participation component of the ICF, especially the Mobility chapter. Nevertheless, Environmental Factors accounted for 35% of the results analyzed, a figure that favorably complements other recent reviews (e.g., [12,83]) and may indicate growing interest in this underexplored component.

Despite these strengths, certain limitations must be acknowledged. The search was restricted to studies published in English or Spanish, which may have excluded relevant findings from other linguistic or regional contexts. Although no age filter was applied, the sample across the studies ended up being composed predominantly of children and adolescents under the age of 20. On the one hand, although the review addressed aspects such as mobility, self-care, or recreation—which are also ICF topics relevant in adults [68]—this does not guarantee that the findings can be extrapolated to the adult population. On the other hand, it implies that other aspects of particular importance to adults, such as employment [68], may not have been addressed, which indirectly represents not only a limitation in scientific terms but also in clinical practice. Additionally, the broad analytical scope adopted in this review enabled a comprehensive overview of the relationships between cognition and ICF components, but made it more challenging to extract specific, conclusive patterns. Combined with the heterogeneity of study designs and sample characteristics—including variation in CP severity, GMFCS, and MACS levels—this underscores the need for further research aimed at clarifying the specific cognitive correlates of ICF components and enhancing the generalizability of findings to the wider CP population. Finally, just over half (61.3%) of the articles included were classified as having an OCEBM level of 4, highlighting the need to conduct studies employing methodologies that increase the quality and rigor of findings in this area.

## 5. Conclusions

In conclusion, this review confirms that cognitive functioning is meaningfully associated with multiple ICF components in individuals with CP. These findings emphasize the crucial role of early and comprehensive cognitive assessments in children with CP to guide individualized interventions across multiple domains, including motor skills, communication, self-care, and social participation. While the Activities and Participation component remains the most frequently assessed domain, this review also highlights an encouraging increase in the representation of Environmental Factors, although this area remains insufficiently explored. Considering the use of assistive technologies, providing family and educational support, and multidisciplinary service provision, tailored to cognitive profiles, is essential to optimize outcomes and ensure holistic care.

Further research is needed to extrapolate these findings to a broader population, considering the heterogeneity of the condition and the possible related conditions. Moreover, there is a clear need to promote the systematization and standardization of both cognitive and ICF-related assessments, with special emphasis on adaptability for individuals with severe impairments. Such efforts will not only improve data comparability across studies but also support more inclusive, comprehensive, and person-centred approaches to assessment and intervention in CP.

## Figures and Tables

**Table 1:** Keywords used in the search.

Population	Neuropsychology/Cognition	ICF Components ^1^
cerebral palsy	neuropsycholog*, cogniti*, intelligence, intellectual, executive func*, language, memory, verbal learning, nonverbal learning, visual percep*, visuospatial ability, spatial processing, attention, cognitive processing speed, social cognition, theory of mind, emotion recognition	literacy, reading, writing, math*, learning, activities of daily living, nonverbal communication, augmentative communication, motor activity, physical mobility, household management, household work, interpersonal relations, interpersonal interaction, interpersonal relationships, education, work, employment, participation, social participation, community participation, community involvement, leisure activities, recreation, technology, social support, community support, attitude, social services, policy, international classification of functioning, disability and health

Abbreviations: ICF, International Classification of Functioning, Disability and Health. ^1^ ICF components of Activities and Participation and Environmental Factors. *, truncation symbol used to retrieve all word variants.

**Table 8:** Major Life Areas (d8).

ICF		Cognitive Assessment	Demographic Data	Main Results *
**ICF chapter**	**ICF second level** *Assessment*	**Cognitive domain** *Instrument*	n Age range (years:months) n females n type CP n pattern CP Motor ability	**+ (significative)** **− (significative)** **n.s.** Author (year)—LOE
**d8 Major life areas**	**d820 School education** *School Function Assessment (SFA)*	**Memory** *School Function Assessment (SFA)*	148 6:1–13:6 years 61 females CP type unk CP pattern unk GMFCS: 148 II–IV	**+ (significative)** Schenker et al. (2005) [51]—LOE 4

Abbreviations: − (significative), negative tendency with significative results; + (significative), positive tendency with significative results; CP, cerebral palsy; GMFCS, Gross Motor Function Classification System; ICF, International Classification of Functioning, Disability and Health; LOE, level of evidence; n.s., no significative results; unk, unknown; italic format, measures used; *, detailed results are presented in Supplementary Table S3.

## Data Availability

The reviewed data can be reasonably shared upon request by contacting the corresponding author.

## Supplementary Materials

The following supporting information can be downloaded at: https://www.mdpi.com/article/10.3390/jcm14186393/s1, Table S1: Full search strategy; Table S2: Updated full search strategy; Table S3: Detailed information of studies examining the relationship between cognitive functioning and ICF components (excluding intervention studies); Table S4: Detailed information of articles exploring the effects of health interventions on cognition; Table S5: Detailed information of articles exploring the effects of cognitive interventions on non-cognitive aspects of functioning.
