# Supplementary material for: The Role of Cognitive Functioning in the ICF Framework: A Systematic Review of Its Influence on Activities and Participation and Environmental Factors in People with Cerebral Palsy
Source: J Clin Med. 2025 Sep 10;14(18):6393. doi: 10.3390/jcm14186393 (PMC12470702; doi:10.3390/jcm14186393)
Supplement: Supplementary file 1 [file jcm-14-06393-s001.zip › Supplementary Table S2.pdf]

Supplementary Table S2. Updated full search strategy.

Date of the search: 2nd April 2025 (search until 31st March 2025)

Database	Search equation	Filters applied	Results
PubMed	("cerebral palsy"[MeSH Terms] AND ("neuropsycholog*"[MeSH Terms] OR "cogniti*"[MeSH Terms] OR "intelligence"[MeSH Terms] OR "executive func*"[MeSH Terms] OR "language"[MeSH Terms] OR "memory"[MeSH Terms] OR "verbal learning"[MeSH Terms] OR "visual percep*"[MeSH Terms] OR "spatial processing"[MeSH Terms] OR "attention"[MeSH Terms] OR "social cognition"[MeSH Terms] OR "theory of mind"[MeSH Terms]) AND ("literacy"[MeSH Terms] OR "reading"[MeSH Terms] OR "writing"[MeSH Terms] OR "math*"[MeSH Terms] OR "learning"[MeSH Terms] OR "activities of daily living"[MeSH Terms] OR "nonverbal communication"[MeSH Terms] OR "motor activity"[MeSH Terms] OR "Household work"[MeSH Terms] OR "Interpersonal Relations"[MeSH Terms] OR "education"[MeSH Terms] OR "work"[MeSH Terms] OR "employment"[MeSH Terms] OR "social participation"[MeSH Terms] OR "community participation"[MeSH Terms] OR "leisure activities"[MeSH Terms] OR "recreation"[MeSH Terms] OR "technology"[MeSH Terms] OR "social support"[MeSH Terms] OR "community support"[MeSH Terms] OR "attitude"[MeSH Terms] OR "policy"[MeSH Terms] OR "international classification of functioning, disability and health"[MeSH Terms]))	AND ((casereports[Filter] OR classicalarticle[Filter] OR clinicalstudy[Filter] OR clinicaltrial[Filter] OR clinicaltrialphasei[Filter] OR clinicaltrialphaseii[Filter] OR clinicaltrialphaseiii[Filter] OR clinicaltrialphaseiv[Filter] OR comparativestudy[Filter] OR consensusdevelopmentconference[Filter] OR consensusdevelopmentconferencenih[Filter] OR controlledclinicaltrial[Filter] OR correctedandrepublishedarticle[Filter] OR evaluationstudy[Filter] OR guideline[Filter] OR historicalarticle[Filter] OR meta-analysis[Filter] OR multicenterstudy[Filter] OR observationalstudy[Filter] OR practiceguideline[Filter] OR pragmaticclinicaltrial[Filter] OR publishederratum[Filter] OR randomizedcontrolledtrial[Filter] OR researchsupportamericanrecoveryandreinvestmentact[Filter] OR researchsupportnihextramural[Filter] OR researchsupportnihintramural[Filter] OR researchsupportnonusgovt[Filter] OR researchsupportusgovtnonphs[Filter] OR researchsupportusgovtphs[Filter] OR researchsupportusgovernment[Filter] OR review[Filter] OR systematicreview[Filter] OR technicalreport[Filter] OR twinstudy[Filter] OR validationstudy[Filter]) AND (humans[Filter]) AND (2023/5/4:2025/3/31[pdat]))	16
WOS	((AK=("cerebral palsy")) AND AK=("neuropsycholog*" OR "cogniti*" OR "intelligence" OR "executive func*" OR "language" OR "memory" OR "verbal learning" OR "visual percep*" OR "spatial processing" OR "attention" OR "social cognition" OR "theory of mind")) AND AK=("literacy" OR "reading" OR "writing" OR "math*" OR "learning" OR "activities of daily living" OR "nonverbal communication" OR "motor activity" OR "Household work" OR "Interpersonal Relations" OR "education" OR "work" OR "employment" OR "social participation" OR "community participation" OR "leisure activities" OR "recreation" OR "technology" OR "social support" OR "community support" OR "attitude" OR "policy" OR "international classification of functioning, disability and health")	and Timespan: 2023-05-04 to 2025-03-31 (Index Date)	6
PsycINFO	(subject("cerebral palsy") AND (subject("neuropsycholog*") OR subject("cogniti*") OR subject("intelligence") OR subject ("intellectual") OR subject(("executive function" OR "executive functioning" OR "executive functions")) OR subject("language") OR subject("memory") OR subject("verbal learning") OR subject("nonverbal learning") OR subject(("visual perception" OR "visual perceptions" OR "visual percepts" OR "visual perceptual")) OR subject("visuospatial ability") OR subject("spatial processing") OR subject("attention") OR subject("cognitive processing speed") OR subject("social cognition") OR subject("theory of mind") OR subject("emotion recognition")) AND (subject("literacy") OR subject("reading") OR subject("writing") OR subject("math*") OR subject("learning") OR	AND po.exact("male" OR "transgender" OR "inpatient" OR "outpatient" OR "human" OR "female") AND PEER(yes) AND Publication date (04/05/2023 – 31/03/2025)	16

	subject("activities of daily living") OR subject("nonverbal communication") OR subject("augmentative communication") OR subject("motor activity") OR subject("physical mobility") OR subject("Household Management") OR subject("Household work") OR subject("Interpersonal Relations") OR subject("Interpersonal Interaction") OR subject("interpersonal Relationships") OR subject("education") OR subject("work") OR subject("employment") OR subject("participation") OR subject("social participation") OR subject("community participation") OR subject("community involvement") OR subject("leisure activities") OR subject("recreation") OR subject("technology") OR subject("social support") OR subject("community support") OR subject("attitude") OR subject("social services") OR subject("policy") OR subject("International Classification of Functioning, Disability and Health")))		
CENTRAL	(cerebral palsy):kw AND (neuropsycholog* OR cogniti* OR "intelligence" OR executive func* OR "language" OR "memory" OR "verbal learning" OR visual percep* OR "spatial processing" OR "attention" OR "social cognition" OR "theory of mind"):kw AND ("literacy" OR "reading" OR "writing" OR math* OR "learning" OR "activities of daily living" OR "nonverbal communication" OR "motor activity" OR "Household work" OR "Interpersonal Relations" OR "education" OR "work" OR "employment" OR "social participation" OR "community participation" OR "leisure activities" OR "recreation" OR "technology" OR "social support" OR "community support" OR "attitude" OR "policy" OR "international classification of functioning, disability and health"):kw	with Cochrane Library publication date Between May 2023 and Mar 2025	7
CINAHL	SU "cerebral palsy" AND SU ("neuropsycholog*" OR "cogniti*" OR "intelligence" OR "executive func*" OR "language" OR "memory" OR "verbal learning" OR "visual percep*" OR "spatial processing" OR "attention" OR "social cognition" OR "theory of mind") AND SU ("literacy" OR "reading" OR "writing" OR "math*" OR "learning" OR "activities of daily living" OR "nonverbal communication" OR "motor activity" OR "Household work" OR "Interpersonal Relations" OR "education" OR "work" OR "employment" OR "social participation" OR "community participation" OR "leisure activities" OR "recreation" OR "technology" OR "social support" OR "community support" OR "attitude" OR "policy" OR "international classification of functioning, disability and health")	Publication date: 20230504-20250331; Peer-review; Humans	25
ERIC	(subject("cerebral palsy") AND (subject("neuropsycholog*") OR subject("cogniti*") OR subject("intelligence") OR subject ("intellectual") OR subject(("executive function" OR "executive functioning" OR "executive functions")) OR subject("language") OR subject("memory") OR subject("verbal learning") OR subject("nonverbal learning") OR subject(("visual perception" OR "visual perceptions" OR "visual percepts" OR "visual perceptual")) OR subject("visuospatial ability") OR subject("spatial processing") OR subject("attention") OR subject("cognitive processing speed") OR subject("social cognition") OR subject("theory of mind") OR subject("emotion recognition")) AND (subject("literacy") OR subject("reading") OR subject("writing") OR subject("math*") OR subject("learning") OR subject("activities of daily living") OR subject("nonverbal communication") OR subject("augmentative communication") OR subject("motor activity") OR	AND (pd(20230504-20250331) AND PEER(yes))	3

	subject("physical mobility") OR subject("Household Management") OR subject("Household work") OR subject("Interpersonal Relations") OR subject("Interpersonal Interaction") OR subject("interpersonal Relationships") OR subject("education") OR subject("work") OR subject("employment") OR subject("participation") OR subject("social participation") OR subject("community participation") OR subject("community involvement") OR subject("leisure activities") OR subject("recreation") OR subject("technology") OR subject("social support") OR subject("community support") OR subject("attitude") OR subject("social services") OR subject("policy") OR subject("International Classification of Functioning, Disability and Health")))

Abbreviations: CENTRAL, Central Register of Controlled Trials; CINAHL, Cumulative Index to Nursing and Allied Health Literature: ERIC, Education Resources Information Center; WOS, Web of Science.
