# Supplementary material for: The Role of Cognitive Functioning in the ICF Framework: A Systematic Review of Its Influence on Activities and Participation and Environmental Factors in People with Cerebral Palsy
Source: J Clin Med. 2025 Sep 10;14(18):6393. doi: 10.3390/jcm14186393 (PMC12470702; doi:10.3390/jcm14186393)
Supplement: Supplementary file 1 [file jcm-14-06393-s001.zip › Supplementary Table S3.pdf]

**Supplementary Table S3.** Detailed information of studies examining the relationship between cognitive functioning and ICF components (excluding intervention studies).

| Reference | Participants | ICF Assessment | Cognitive Assessment | Outcomes |
|---|---|---|---|---|
| Authors (year) [reference] | n<br>Age range (years:months)<br>n females<br>n type CP<br>n pattern CP<br>Motor ability | **ICF component**<br>ICF chapter; ICF second level<br>*Assessment*: subscales | **Cognitive domain**<br>*Instrument*: subscales | **Statistical method**<br>Cognitive assessment x ICF assessment<br>Results |
| Falkman et al. (2002) [30] | 7<br>5:1-7:3 years<br>5 females<br>3 spastic, 2 dyskinetic, 1 ataxic, 1 other<br>1 unilateral, 2 bilateral, 4 unk<br>Motor ability unk | **d Activities and Participation**<br>d3 Communication; d331 Non-speech vocal expression, d335 Producing nonverbal messages<br>*The material used was part of a larger study on communication in non-vocal children with cerebral palsy (Dahlgren Sandberg and Hjelmquist 1996)* | **General intellectual functioning**<br>*Raven Coloured Progressive Matrices (RCPM)*<br>**Language**<br>*Språkligt Impressivt Test (SIT)*<br>*Syntactic acceptability:* judgement and correction | **Correlation**<br>RCPM x Communication modes: r = n.s.<br>SIT x Communication modes: r = n.s.<br>Syntactic acceptability x Communication modes: r = n.s. |
| Dellatolas et al. (2005) [44] | 30<br>7:0-8:0 years<br>12 females<br>29 spastic, 1 mixed<br>20 unilateral, 10 bilateral<br>Motor ability unk | **d Activities and Participation**<br>d4 Mobility; d440 Fine hand use<br>*Computerised Peg Moving Task (CPMT)* | **General intellectual functioning**<br>*Raven Coloured Progressive Matrices (RCPM)*<br>**Language/Visual perception**<br>*Neuropsychological battery:* semantic fluency, visual recognition, phonological fluency, digit span, span for words, span for nonwords, figure reproduction, embedded figure, counting dots, word list recall | **Correlation**<br>*Bilateral group:*<br>RCPM x CPMT time (best hand): r = -0.77*<br>*Unilateral group:*<br>RCPM x CPMT time (best hand): n.s.<br>*Total:*<br>RCPM x CPMT time (best hand): r = -0.225<br>Neuropsychological battery x CPMT time (best hand)<br>Semantic fluency: r = 0.038<br>Visual recognition: r = -0.025<br>Phonological fluency: r = 0.046<br>Digit span: r = -0.086<br>Span for words: r = -0.083<br>Span for nonwords: r = -0.115<br>Figure reproduction: r = -0.442*<br>Embedded figure: r = -0.528**<br>Counting dots: r = -0.557**<br>Word list recall: r = -0.222 |
| Schenker et al. (2005) [51] | 148 (FI: 100, SC: 48)<br>6:1-13:6 years<br>61 females<br>CP type unk | **d Activities and Participation**<br>d8 Major life areas; d820 School education | **Memory**<br>*School Function Assessment (SFA):* activity performance (memory and understanding) | **Correlation**<br>SFA x SFA<br>*FI group:* |

| | | | | |
|---|---|---|---|---|
| | CP pattern unk<br>GMFCS: 148 II-IV | *School Function Assessment (SFA):* participation | | Activity performance (memory and understanding) x Participation: r = 0.23*<br>*SC group:*<br>Activity performance (memory and understanding) x Participation: r = 0.41** |
| Pirila et al. (2007) [46] | 36<br>1:10-9:0 years<br>16 females<br>22 spastic, 14 unk<br>5 unilateral, 31 bilateral<br>GMFCS: 7 III, 12 I-II, 17 IV-V | **d Activities and Participation**<br>d4 Mobility; d450-d469 Walking and moving<br>*Gross Motor Limitation Scale*<br>e **Environmental Factors**<br>e1 Products and technology; e125 Products and technology for communication<br>*AAC*<br>e5 Services, systems and policies; e580 Health services, systems and policies<br>*Speech therapy* | **General intellectual functioning**<br>*Wechsler Preschool and Primary Scales of Intelligence-Revised (WPPSI-R)*<br>*Wechsler Intelligence Scale for Children-Revised (WISC-R)*<br>*Griffiths Scales of Mental Development (GSMD)*<br>**Language**<br>*Reynell Developmental Language Scale-Revised (RDLS-R)*<br>*Bo Ege Test Verbal Language Development Scale* | **Chi-square**<br>RDLS-R x Gross Motor Limitation Scale: $\chi^2 = 13.31$**<br>Bo Ege Test Verbal Language Development Scale x Gross Motor Limitation Scale: $\chi^2 = 14.31$**<br>WPPSI-R, WISC-R, GSMD x AAC: $\chi^2 = 13.11$**<br>WPPSI-R, WISC-R, GSMD x Speech therapy: $\chi^2 = 3.94$* |
| Majnemer et al. (2008) [52] | 67<br>6:1-12:11 years<br>25 females<br>56 spastic, 11 other<br>23 unilateral, 33 bilateral, 11 other<br>GMFCS: 40 I, 15 II, 12 III–V | **d Activities and Participation**<br>d9 Community, social and civic life; d920 Recreation and leisure<br>*Children's Assessment of Participation and Enjoyment (CAPE)* | **General intellectual functioning**<br>*Leiter International Performance Scale-Revised (Leiter-R)* | **Multivariate regression**<br>CAPE (diversity informal activities) x IQ (composite) + GMFM: p (model) = < 0.002; $r^2$ (variance) = 21%<br>IQ (composite): parameter estimate = 0.08**<br>CAPE (intensity informal activities) x IQ (composite) + Family impact (health condition): p (model) = < 0.001; $r^2$ (variance) = 26%<br>IQ (composite): parameter estimate = 0.01*<br>CAPE (enjoyment informal activities) x IQ (composite) + Parental stress + Sex + Age: p (model) = < 0.001; $r^2$ (variance) = 46%<br>IQ (composite): parameter estimate = n.a.*<br>CAPE (diversity social) x IQ (composite) + mastery pleasure: p (model) = < 0.001; $r^2$ (variance) = 36%<br>IQ (composite): parameter estimate = 0.03**<br>CAPE (enjoyment recreational) x IQ (composite) + behaviour problems (peer relations) + parental stress (difficult child): p (model) = 0.006; $r^2$ (% variance) = 25%<br>IQ (composite): parameter estimate = -0.01 |
| Cunningham et al. (2009) [49] | 41<br>6:0-12:0 years | **d Activities and Participation** | **General intellectual functioning** | **Correlation**<br>WISC-III x FQQ |

| | | | | |
|---|---|---|---|---|
| | 18 females<br>38 spastic, 3 unk<br>10 unilateral, 28 bilateral, 3 unk<br>GMFCS: 5 I, 1 II, 17 III, 5 IV, 1 V, 12 unk | d7 Interpersonal interactions and relationships; d710 Basic interpersonal interactions<br>*Friendship Quality Questionnaire (FQQ):* validation & caring, conflict & betrayal subscales<br>*Personality Inventory for Children-2nd edition (PIC-2):* social adjustment<br>**e Environmental Factors**<br>e3 Support and relationships; e320 Friends<br>*Social Network Inventory for Children (SNIC):* number of friends<br>e4 Attitudes; e410 Individual attitudes of immediate family members<br>*Parenting Dimensions Inventory (PDI):* nurturance, nonrestrictive attitude, organization | *Wechsler Intelligence Scale for Children-3rd edition (WISC-III):* vocabulary subtest | Validation & caring: r = 0.16<br>Conflict & betrayal: r = 0.10<br>WISC-III x PIC-2: r = -0.36*<br>WISC-III x SNIC: r = 0.37*<br>WISC-III x PDI<br>Nurturance: r = 0.17<br>Nonrestrictive attitude: r = -0.18<br>Organization: r = 0.10 |
| Peeters et al. (2009) [26] | 40<br>72m ± 5.8m<br>17 females<br>35 spastic, 1 ataxic, 4 mixed<br>8 unilateral, 31 bilateral, 1 unk<br>Motor ability unk | **d Activities and Participation**<br>d1 Learning and applying knowledge; d166 Reading<br>*Five parent questionnaires regarding Home Literacy Variables:* child literacy interest, child's activities during storybook reading, materials and parental activities for child literacy development, parents' literacy materials and activities, parents' expectations for their child's literacy development | **General intellectual functioning**<br>*Raven Coloured Progressive Matrices (RCPM)*<br>**Language**<br>*Peabody Picture Vocabulary Test-3rd edition (PPVT-III)* | **Correlation**<br>RCPM x Parent questionnaire<br>Child writing experiences: r = 0.24<br>Child experiences with literacy materials: r = 0.16<br>Child storybook reading interest: r = -0.03<br>Child story orientation activities: r = 0.14<br>Child word orientation activities: r = 0.40*<br>Child book orientation activities: r = 0.30<br>Provision of literacy materials: r = 0.09<br>Parent storybook reading: r = -0.20<br>Parent literacy mediation: r = 0.32*<br>Parent leisure activities: r = 0.11<br>Parent book reading: r = 0.15<br>Parent e-mail use: r = 0.28<br>Parent magazine reading: r = -0.01<br>PPVT-III x Parent questionnaire<br>Child writing experiences: r = 0.29<br>Child experiences with literacy materials: r = 0.23<br>Child storybook reading interest: r = 0.04<br>Child story orientation activities: r = 0.19 |

| | | | | |
|---|---|---|---|---|
| | | | | Child word orientation activities: r = 0.26<br>Child book orientation activities: r = 0.04<br>Provision of literacy materials: r = 0.04<br>Parent storybook reading: r = 0.02<br>Parent literacy mediation: r = 0.30<br>Parent leisure activities: r = -0.04<br>Parent book reading: r = 0.04<br>Parent e-mail use: r = 0.03<br>Parent magazine reading: r = -0.10 |
| Peeters et al. (2009) [41] | 49<br>71.88m ± 5.82m<br>18 females<br>48 spastic, 1 ataxic<br>7 unilateral, 40 bilateral, 2 unk<br>Motor ability unk | **d Activities and Participation**<br>d4 Mobility; d430-d449 Carrying, moving and handling objects<br>*Manual Ability Classification System (MACS)*<br>d410-d429 Changing and maintaining body position; d450-d469 Walking and moving<br>*Gross Motor Function Classification System (GMFCS)*<br>**e Environmental Factors**<br>e4 Attitudes; e430 Individual attitudes of people in positions of authority<br>*Teacher reading expectations*<br>*Teacher writing expectations* | **General intellectual functioning**<br>*Raven Coloured Progressive Matrices (RCPM)*<br>**Language**<br>*Dutch Language Proficiency Test:* syntactic pattern<br>*Peabody Picture Vocabulary Test-3rd edition (PPVT-III)* | **Correlation**<br>*Teacher reading expectations:*<br>RCPM x Reading expectation: r = 0.61**<br>RCPM x MACS: r = -0.13<br>RCPM x GMFCS: r = -0.25<br>Dutch Language Proficiency Test, PPVT-III x Reading expectation: r = 0.55**<br>Dutch Language Proficiency Test, PPVT-III x MACS: r = -0.43**<br>Dutch Language Proficiency Test, PPVT-III x GMFCS: r = -0.44**<br>*Teacher writing expectations:*<br>RCPM x Writing expectation: r = 0.7**<br>RCPM x MACS: r = -0.07<br>RCPM x GMFCS: r = -0.23<br>Dutch Language Proficiency Test, PPVT-III x Writing expectation: r = 0.31<br>Dutch Language Proficiency Test, PPVT-III x MACS: r = -0.34*<br>Dutch Language Proficiency Test, PPVT-III x GMFCS: r = -0.28 |
| Peeters et al. (2009) [27] | 35<br>6:0-7:0 years<br>21 females<br>34 spastic, 1 ataxic<br>5 unilateral, 25 bilateral, 5 unk<br>Motor ability unk | **d Activities and Participation**<br>d1 Learning and applying knowledge; d166 Reading<br>*Four self-administrated parent questionnaires:* child literacy interest, child's activities during storybook reading, materials and parental activities for child literacy development, and parent's own literacy materials and activities | **Language**<br>*Peabody Picture Vocabulary Test-3rd edition (PPVT-III)*<br>*Dutch Language Proficiency Test:* syntactic pattern<br>*Dutch Specific Language Impairment (SLI):* Screening Test Battery, standardized subtest for letter knowledge<br>*Reading Technology Test, shortened version* | **Correlation**<br>*Time 1:*<br>PPVT-III x Parent questionnaire<br>Child writing experiences: r = 0.12<br>Child experiences of literacy materials: r = 0.14<br>Child storybook reading: r = 0.07<br>Child story orientation activities: r = 0.31<br>Child word orientation activities: r = 0.37*<br>Child book orientation activities: r = 0.08<br>Provision of literacy materials: r = -0.12<br>Parent storybook reading: r = -0.07<br>Parent literacy mediation: r = 0.49**<br>Parent leisure activities: r = 0.12 |

Parent own book reading: r = -0.11
Parent email use: r = -0.11
Parent magazines reading: r = -0.18
<u>Dutch Language Proficiency Test x Parent questionnaire</u>
Child writing experiences: r = 0.38*
Child experiences of literacy materials: r = 0.27
Child storybook reading: r = 0.09
Child story orientation activities: r = 0.31
Child word orientation activities: r = 0.29
Child book orientation activities: r = 0.35*
Provision of literacy materials: r = 0.14
Parent storybook reading: r = -0.02
Parent literacy mediation: r = 0.55**
Parent leisure activities: r = -0.02
Parent own book reading: r = 0.19
Parent email use: r = 0.20
Parent magazines reading: r = -0.10
<u>SLI x Parent questionnaire</u>: r = n.a.
<u>Reading Technology Test x Parent questionnaire</u>: r = n.a.
*Time 2:*
<u>PPVT-III x Parent questionnaire</u>
Child writing experiences: r = 0.14
Child experiences of literacy materials: r = 0.11
Child storybook reading: r = 0.12
Child story orientation activities: r = 0.29
Child word orientation activities: r = 0.30
Child book orientation activities: r = 0.00
Provision of literacy materials: r = -0.10
Parent storybook reading: r = 0.01
Parent literacy mediation: r = 0.49**
Parent leisure activities: r = 0.00
Parent own book reading: r = -0.13
Parent email use: r = 0.01
Parent magazines reading: r = -0.07
<u>Dutch Language Proficiency Test x Parent questionnaire</u>
Child writing experiences: r = 0.19
Child experiences of literacy materials: r = 0.01
Child storybook reading: r = 0.13
Child story orientation activities: r = 0.51**

Child word orientation activities: r = 0.37*
Child book orientation activities: r = 0.09
Provision of literacy materials: r = -0.12
Parent storybook reading: r = 0.05
Parent literacy mediation: r = 0.50**
Parent leisure activities: r = -0.11
Parent own book reading: r = -0.01
Parent email use: r = 0.00
Parent magazines reading: r = -0.07
SLI x Parent questionnaire
Child writing experiences: r = 0.14
Child experiences of literacy materials: r = 0.18
Child storybook reading: r = 0.25
Child story orientation activities: r = 0.54**
Child word orientation activities: r = 0.51**
Child book orientation activities: r = 0.12
Provision of literacy materials: r = 0.02
Parent storybook reading: r = 0.16
Parent literacy mediation: r = 0.70**
Parent leisure activities: r = 0.36*
Parent own book reading: r = 0.09
Parent email use: r = 0.04
Parent magazines reading: r = -0.13
Reading Technology Test x Parent questionnaire
Child writing experiences: r = 0.18
Child experiences of literacy materials: r = 0.25
Child storybook reading: r = 0.19
Child story orientation activities: r = 0.53**
Child word orientation activities: r = 0.43**
Child book orientation activities: r = 0.20
Provision of literacy materials: r = 0.11
Parent storybook reading: r = 0.25
Parent literacy mediation: r = 0.62**
Parent leisure activities: r = 0.36*
Parent own book reading: r = 0.12
Parent email use: r = 0.02
Parent magazines reading: r = -0.12
**Multiple regression**
*Reading precursors, time 1:*

| | | | | |
|---|---|---|---|---|
| | | | | PPVT-III x Parent questionnaire<br>Parent literacy mediation: $r^2$ = 0.15*<br>Child story orientation activities: $r^2$ = 0.8<br>Child word orientation activities: $r^2$ = 0.6<br>Dutch Language Proficiency Test x Parent questionnaire<br>Parent literacy mediation: $r^2$ = 0.30**<br>Child story orientation activities: $r^2$ = 0.10<br>Child word orientation activities: $r^2$ = 0.09<br>*Early reading skills:*<br>SLI x Parent questionnaire<br>Parent literacy mediation: $r^2$ = 0.49**<br>Child story orientation activities: $r^2$ = 0.29**<br>Child word orientation activities: $r^2$ = 0.26**<br>Reading Technology Test x Parent questionnaire<br>Parent literacy mediation: $r^2$ = .38**<br>Child story orientation activities: $r^2$ = 0.28**<br>Child word orientation activities: $r^2$ = 0.19**<br>**Incremental $R^2$ of the hierarchical multiple regression**<br>SLI x Parent questionnaire<br>Parent literacy mediation: $r^2$ = 0.04<br>Child story orientation: $r^2$ = 0.00<br>Child word orientation: $r^2$ = 0.03<br>Reading Technology Test x Parent questionnaire<br>Parent literacy mediation: $r^2$ = 0.03<br>Child story orientation: $r^2$ = 0.01<br>Child word orientation: $r^2$ = 0.01 |
| Holck et al. (2010) [31] | 10<br>6:0-10:6 years<br>3 females<br>10 spastic<br>10 unilateral<br>Motor ability unk | **d Activities and Participation**<br>d3 Communication; d310-d329 Communicating - receiving<br>*Children's Communication Checklist (CCC)*: 5 subscales constituting the pragmatic composite | **Language**<br>*Material from Bishop and Adams (1992; translated and adapted to Swedish by the authors):* literal and inferential comprehension | **Partial correlation with mental age as a covariate**<br>Literal comprehension x CCC: r = 0.889*<br>Inferential comprehension x CCC: n.s. |
| Majnemer et al. (2010) [53] | 55<br>6:1-12:11 years<br>19 females<br>48 spastic, 7 unk<br>18 unilateral, 30 bilateral, 7 unk | **d Activities and Participation**<br>d9 Community, social and civic life; d920 Recreation and leisure<br>*Preferences for Activities of Children (PAC)* | **General intellectual functioning**<br>*Leiter International Performance Scale-Revised (Leiter-R)* | **Multiple linear regression**<br>PAC (active-physical) x age at assessment + Dimensions of Mastery Questionnaire (gross motor persistence) + Leiter-R: $r^2$ (% variance) = 39.1**<br>Leiter-R: parameter estimate: beta = 0.007; 95% CI = 0.001-0.01 |

| | | | | |
|---|---|---|---|---|
| | GMFCS: 34 I, 12 II, 4 V, 5 III-IV | | | |
| Peeters et al. (2011) [25] | 52<br>5:0-6:3 years<br>19 females<br>43 spastic, 2 ataxic, 7 mixed<br>8 unilateral, 35 bilateral, 9 unk<br>Motor ability unk | **d Activities and Participation**<br>d1 Learning and applying knowledge; d166 Reading<br>*Questionnaire about emergent literacy activities* | **General intellectual functioning**<br>*Raven Coloured Progressive Matrices (RCPM)*<br>**Language**<br>*Dutch Language Proficiency Test:* syntactic pattern<br>*Peabody Picture Vocabulary Test-3rd edition (PPVT-III)* | **Correlation**<br>PPVT-III x Teacher questionnaire<br>Storybook reading, time: r = 0.11<br>Rhyming games and singing, time: r = 0.12<br>Independent picture-book reading, time: r = 0.03<br>Educational software, time: r = -0.05<br>Training reading precursors, time: r = 0.49**<br>Amount of reading precursors trained, total: r = 0.36*<br>Instruction outside classroom: r = -0.07<br>Amount of therapy: r = -0.22<br>Missing instruction cause of therapy: r = 0.04<br>School absence: r = -0.10<br>Dutch Language Proficiency Test x Teacher questionnaire<br>Storybook reading, time: r = 0.25<br>Rhyming games and singing, time: r = 0.03<br>Independent picture-book reading, time: r = 0.18<br>Educational software, time: r = 0.00<br>Training reading precursors, time: r = 0.44**<br>Amount of reading precursors trained, total: r = 0.42**<br>Instruction outside classroom: r = -0.06<br>Amount of therapy: r = -0.05<br>Missing instruction cause of therapy: r = 0.06<br>School absence: r = 0.11<br>RCPM x Teacher questionnaire<br>Storybook reading, time: r = 0.44**<br>Rhyming games and singing, time: r = 0.11<br>Independent picture-book reading, time: r = 0.19<br>Educational software, time: r = 0.06<br>Training reading precursors, time: r = 0.33* |

| | | | | |
|---|---|---|---|---|
| | | | | Amount of reading precursors, total: r = 0.34*<br>Instruction outside classroom: r = 0.08<br>Number of adaptations in method: r = -0.23<br>Amount of therapy: r = 0.10<br>Missing instruction cause of therapy: r = -0.06<br>School absence: r = 0.06 |
| Smits et al. (2011) [42] | 116<br>4:8-7:7 years<br>40 females<br>98 spastic, 14 dyskinetic, 4 ataxic<br>42 unilateral, 56 bilateral, 18 unk<br>GMFCS: 56 I, 20 II, 17 III, 9 IV, 14 V<br>MACS: 33 I, 40 II, 14 III, 8 IV, 11 V, 10 unk | **d Activities and Participation**<br>d4 Mobility; d410-d429 Changing and maintaining body position, d450 Walking<br>d5 Self-care; d510 Washing oneself, d520 Caring for body parts, d530 Toileting, d540 Dressing, d550 Eating, d560 Drinking<br>d7 Interpersonal interactions and relationships; d710-d729 General interpersonal interactions<br>*Pediatric Evaluation of Disability Inventory (PEDI):* functional skills scale (FSS) | **General intellectual functioning**<br>*Raven Coloured Progressive Matrices (RCPM)* | **Linear mixed models**<br><u>RCPM (time) x PEDI-FSS</u><br>Self-care: beta = 0.09; SE = 0.01**<br>Social function: beta = 0.03; SE = 0.02*<br>Mobility: beta = 0.03; SE = 0.02<br>**Multivariate model backward stepwise procedure**<br><u>PEDI-FSS (self-care) x GMFCS level + RCPM (IQ score):</u> $r^2$ = 73%<br>RCPM (IQ score): beta = 0.17; SE = 1.06**<br>RCPM (time, IQ score): beta = 0.06; SE = 0.02**<br>**Post hoc**<br>PEDI-FSS (self-care) x RCPM (IQ score): t = 3.42** |
| Dalvand et al. (2012) [36] | 662<br>3:0-14:0 years<br>281 females<br>498 spastic, 53 dyskinetic, 18 ataxic, 63 mixed, 30 other<br>57 unilateral, 441 bilateral, 164 unk<br>GMFCS: 69 I, 114 II, 138 III, 156 IV, 185 V | **d Activities and Participation**<br>d4 Mobility; d410-d429 Changing and maintaining body position, d450-d469 Walking and moving<br>*Gross Motor Function Classification System, Expanded and Revised (GMFCS E&R)* | **General intellectual functioning**<br>*Wechsler preschool and primary scale of intelligence (WPPSI)*<br>*Wechsler Intelligence Scale for Childre-Revised (WISC-R)* | **Ordinal logistic regression, reference category: GMFCS level V**<br><u>WPPSI, WISC-R x GMFCS, I level:</u> estimate = -3.075; SE = 0.328; Wald = 87.786**<br><u>WPPSI, WISC-R x GMFCS, II level:</u> estimate = -2.725; SE = 0.274; Wald = 98.786**<br><u>WPPSI, WISC-R x GMFCS, III level:</u> estimate = -2.343; SE = 0.257; Wald = 83.428**<br><u>WPPSI, WISC-R x GMFCS, IV level:</u> estimate = -1.541; SE = 0.216; Wald = 51.042** |
| Brossard-Racine et al. (2013) [50] | 160<br>12:0-19:0 years<br>65 females<br>119 spastic, 6 dyskinetic, 35 unk | **d Activities and Participation**<br>d7 Interpersonal interactions and relationships; d720 Complex interpersonal interactions | **General intellectual functioning**<br>*Leiter International Performance Scale-Revised (Leiter-R)* | **Correlation**<br><u>Leiter-R x SDQ</u><br>Prosocial behavior: r = 0.31**<br>Hyperactivity: r = -0.35** |

| | | | | |
|---|---|---|---|---|
| | 42 unilateral, 77 bilateral, 41 unk<br>GMFCS: 111 I-III, 49 IV-V<br>MACS: 47 I, 40 II, 73 unk | *Strengths and Difficulties Questionnaire (SDQ)* | | Emotional symptoms: n.s.<br>Conduct problems: n.s.<br>Peer problems: n.s.<br>Total difficulties: n.s. |
| Chen et al. (2013) [38] | 78<br>1:0-5:6 years<br>29 females<br>CP type unk<br>38 bilateral, 40 unk<br>GMFCS: 20 I, 16 II, 11 III, 14 IV, 17 V | **d Activities and Participation**<br>d4 Mobility; d410-d429 Changing and maintaining body position, d450-d469 Walking and moving<br>*Gross Motor Function Classification System (GMFCS)*<br>*Selective Motor Control (SMC)* | **Cognitive development/Language**<br>*Comprehensive Developmental Inventory for Infants and Toddlers (CDIIT):* cognition, language | **Correlation**<br><u>CDIIT (cognition) x GMFCS:</u> r = -0.256<br><u>CDIIT (cognition) x SMC:</u> r = 0.086<br>**Forward stepwise multipe regression**<br><u>CDIIT (language) x age+ GMFCS levels:</u> F = 17.09**<br>GMFCS: beta = -0.22; 95% CI = -1.08--0.08; adjusted $r^2$ = 0.30 |
| Song (2013) [37] | 68<br>0:1->3:7 years<br>31 females<br>59 spastic, 9 dyskinetic<br>14 unilateral, 45 bilateral<br>Motor ability unk | **d Activities and Participation**<br>d4 Mobility; d410-d429 Changing and maintaining body position, d430-d449 Carrying, moving and handling objects, d450-d469 Walking and moving<br>*Gross Motor Function Measure (GMFM)*<br>*Bayley Infant Development Screening Test-2nd edition (BSID-II):* motor scale | **Cognitive development**<br>*Bayley Infant Development Screening Test-2nd edition (BSID-II):* cognitive (mental) scale | **Correlation**<br><u>BSID-II (cognitive scale) x GMFM:</u> r = 0.719**<br><u>BSID-II (cognitive scale) x BSID-II (motor scale):</u> r = 0.866** |
| Majnemer et al. (2014) [56] | 258<br>6:0-19:0 years<br>102 females<br>CP type unk<br>CP pattern unk<br>GMFCS: 94 I, 8 IV, 24 V, 71 II-III, 53 IV-V, 8 unk | **e Environmental Factors**<br>e5 Services, systems and policies; e580 Health services, systems and policies<br>*Formal questionnaire regarding current educational and rehabilitation services* | **General intellectual functioning**<br>*Leiter International Performance Scale-Revised (Leiter-R)* | **T-test (yes; no)**<br><u>Leiter-R x Service</u><br>*School age:*<br>Occupational therapy: 73.5 ± 20.2; 86.0 ± 20.9*<br>Physical therapy: 75.6 ± 19.1; 88.3 ± 22.2*<br>Speech language pathology: 70.8 ± 5.0; 85.0 ± 3.2<br>Psychology: 82.9 ± 27.2; 80.8 ± 20.6<br>Special education: 76.0 ± 20.9; 83.7 ± 21.4*<br>*Adolescents:*<br>Occupational therapy: 59.8 ± 22.1; 72.8 ± 24.5**<br>Physical therapy: 60.6 ± 23.6; 73.8 ± 23.5**<br>Speech language pathology: 44.9 ± 12.0; 72.3 ± 23.7** |

| | | | | |
|---|---|---|---|---|
| | | | | Psychology: 56.2 ± 15.3; 69.8 ± 25.1**<br>Special education: 52.3 ± 16.3; 74.6 ± 24.3** |
| Muriel et al. (2014) [40] | 15<br>7:0-14:0 years<br>7 females<br>14 spastic, 1 ataxic<br>7 unilateral, 8 bilateral<br>GMFCS: 6 I, 4 II, 2 III, 3 V | **d Activities and Participation**<br>d4 Mobility; d410-d429 Changing and maintaining body position, d450-d469 Walking and moving<br>*Gross Motor Function Classification System (GMFCS)* | **General intellectual functioning/Executive functions**<br>*Wechsler Intelligence Scale for Children-4th edition (WISC-IV):* verbal comprehension index (VCI), processing speed index (PSI), global IQ / perceptual reasoning index (PRI) and working memory index (WMI) | **Kruskall-Wallis**<br>WISC-IV (VCI) x GMFCS: n.s.<br>WISC-IV (PSI) x GMFCS: n.s.<br>WISC-IV (global IQ) x GMFCS: n.s.<br>WISC-IV (PRI) x GMFCS: n.s.<br>WISC-IV (WMI) x GMFCS: n.s. |
| Whittingham et al. (2014) [28] | 46<br>8:0-16:0 years<br>21 females<br>CP type unk<br>46 unilateral<br>GMFCS: 35 I, 11 II<br>MACS: 6 I, 40 II | **d Activities and Participation**<br>d2 General tasks and demands; d230 Carrying out daily routine<br>*Behavior Rating Inventory of Executive Function (BRIEF):* behavioral regulation index (BRI)<br>d7 Interpersonal interactions and relationships; d720 Complex interpersonal interactions<br>*Strengths and Difficulties Questionnaire (SDQ)* | **Executive functions**<br>*Rey Complex Figure Test (RCFT)*<br>*Delis-Kaplan Executive Function System (D-KEFS)*<br>*Test of Every-day Attention for Children (TEA-Ch)*<br>*Wechsler Intelligence Scale for children-4th edition (WISC-IV)* | **Correlation**<br>EF composite x BRIEF-teacher<br>BRI: r = -0.24<br>EF composite x BRIEF-parent<br>BRI: r = -0.41**<br>EF composite x SDQ<br>Emotion: r = 0.10<br>Conduct: r = -0.43**<br>Hyperactivity: r = -0.49**<br>Peer: r = -0.12<br>Prosocial: r = 0.28* |
| James et al. (2015) [29] | 101<br>8:0-17:0 years<br>50 females<br>101 spastic<br>101 unilateral<br>GMFCS: 45 I, 56 II<br>MACS: 24 I, 76 II, 1 III | **d Activities and Participation**<br>d2 General tasks and demands; d230 Carrying out daily routine<br>d4 Mobility; d410-d429 Changing and maintaining body position, d430-d449 Carrying, moving and handling objects<br>*Assessment of Motor and Process Skills-7th edition (AMPS)* | **Visual perception**<br>*Test of Visual Perceptual Skills (Non-Motor)-3rd edition (TVPS-3)* | **Univariable regression**<br>AMPS (motor) x TVPS-3<br>Overall: r = 0.41; beta = 0.01; 95% CI = 0.007-0.02**<br>Visual discrimination: r = 0.31; beta = 0.04; 95% CI = 0.02-0.07**<br>Visual memory: r = 0.38; beta = 0.05; 95% CI = 0.03-0.08**<br>Visual spatial relationships: r = 0.28; beta = 0.03; 95% CI = 0.01-0.05**<br>Form constancy: r = 0.25; beta = 0.03; 95% CI = 0.01-0.06*<br>Visual sequential memory: r = 0.39; beta = 0.05; 95% CI = 0.03-0.07**<br>Figure-ground: r = 0.30; beta = 0.04; 95% CI = 0.01-0.06**<br>Visual closure: r = 0.34; beta = 0.04; 95% CI = 0.02-0.06** |

| | | | | |
|---|---|---|---|---|
| | | | | AMPS (process) x TVPS-3<br>Overall: r = 0.38; beta = 0.01; 95% CI = 0.006-0.02**<br>Visual discrimination: r = 0.29; beta = 0.04; 95% CI = 0.01-0.07**<br>Visual memory: r = 0.41; beta = 0.06; 95% CI = 0.03-0.08**<br>Visual spatial relationships: r = 0.29; beta = 0.03; 95% CI = 0.01-0.05**<br>Form constancy: r = 0.32; beta = 0.04; 95% CI = 0.02-0.07**<br>Visual sequential memory: r = 0.47; beta = 0.06; 95% CI = 0.04-0.08**<br>Figure-ground: r = 0.40; beta = 0.05; 95% CI = 0.03-0.07**<br>Visual closure: r = 0.48; beta = 0.05; 95% CI = 0.03-0.07**<br>**Multivariable model**<br>AMPS (process) x TVPS-3, visual closure + sequential memory + JTTHF dominant upper limb: $r^2$ = 0.35TVPS-3 sequential memory: Bootstrapping coefficients: b = 0.03; BCa 95% CI = 0.01, 0.6** |
| Nordberg et al. (2015) [34] | 15<br>9:2-12:9 years<br>7 females<br>10 spastic, 2 dyskinetic, 3 ataxic<br>8 unilateral, 2 bilateral, 5 unk<br>GMFCS: 9 I, 1 II, 2 III, 3 IV | **d Activities and Participation**<br>d3 Communication; d330 Speaking<br>*Bus Story Test (BST)*<br>*Narrative Assessment Profile (NAP)* | **Language**<br>*Test for Reception of Grammar-Version 2 (TROG-2)*<br>*Peabody Picture Vocabulary Test-4th edition (PPVT-IV)*<br>**General intellectual functioning**<br>*Raven's Coloured Progressive Matrices (RCPM)*<br>**Memory/Executive functions**<br>*Corsi block-tapping test (CB)*<br>*Wechsler Intelligence Scale for Children-3rd edition (WISC-III):* Digit Span (DS)<br>**Social cognition**<br>*False belief items of 2 story tests: "Kiki and the cat" and "Birthday puppy"* | **Correlation**<br>BST (norm referenced information score) x cognitive assessments<br>TROG-2: r = 0.887**<br>PPVT-IV: r = 0.701**<br>CB forward: r = 0.418<br>CB backward: r = 0.498<br>DS forward: r = 0.301<br>DS backward: r = 0.243<br>False second belief: r = –0.719*<br>RCPM: r = 0.487<br>BST (sentence length) x cognitive assessments<br>TROG-2: r = 0.907**<br>PPVT-IV: r = 0.790**<br>CB forward: r = 0.441 |

| | | | | |
|---|---|---|---|---|
| | | | | CB backward: r = 0.446<br>DS forward: r = 0.615*<br>DS backward: r = 0.123<br>False second belief: r = –0.717*<br>RCPM: r = 0.557*<br>BST (subordinate clauses) x cognitive assessments<br>TROG-2: r = 0.849**<br>PPVT-IV: r = 0.716**<br>CB forward: r = 0.446<br>CB backward: r = 0.372<br>DS forward: r = 0.340<br>DS backward: r = 0.259<br>False second belief: r = –0.667*<br>RCPM: r = 0.475<br>BST (sentence length and subordinate clauses) x cognitive assessments<br>TROG-2, PPVT-IV: r = 0.839**<br>CB forward: r = 0.507<br>CB backwards: r = 0.502<br>DS forward: r = 0.624*<br>DS backwards: r = 0.234<br>RCPM: r = 0.490<br>NAP (total) x cognitive assessments<br>TROG-2, PPVT-IV: r = 0.719**<br>CB forward: r = 0.511<br>CB backwards: r = 0.254<br>DS forward: r = 0.588*<br>DS backwards: r = 0.184<br>RCPM: r = 0.058 |
| Wu et al. (2015) [54] | 80<br>2:0-5:11 years<br>30 females | **d Activities and Participation**<br>d9 Community, social and civic life; d920 Recreation and leisure | **Cognitive development** | **Forward stepwise multiple regression** |

| | | | | |
|---|---|---|---|---|
| | CP type unk<br>15 unilateral, 65 bilateral<br>GMFCS: 23 I, 16 II, 12 III, 12 IV, 17 V | *Assessment of Preschool Children's Participation (APCP)* | *Comprehensive Developmental Inventory for Infants and Toddlers (CDIIT):* CDIIT-Cog | APCP (diversity: social activities) x CDIIT-Cog + CP subtypes: F = 6.6**<br>CDIIT-Cog: beta = -0.3; 95%CI = -0.4--0.1; adjusted $r^2$ = 0.074 |
| Hawe et al. (2020) [47] | 45<br>6:5-19:6 years<br>15 females<br>CP type unk<br>45 unilateral<br>MACS: 11 I, 18 II, 16 unk | **d Activities and Participation**<br>d4 Mobility; d445 Hand and arm use<br>*Object hit and avoid task using the Kinarm exoskeleton robot* | **Attention**<br>*Behavioral Inattention Test (BIT):* conventional | **Correlation**<br>BIT x Robot task<br>Hits Nondominant: r = 0.43**<br>Hits Dominant: r = 0.42**<br>Distractors Nondominant: r = −0.03<br>Distractors Dominant: r = −0.41** |
| Milićević (2020) [48] | 110<br>7:0-18:0 years<br>49 females<br>77 spastic, 12 dyskinetic, 11 ataxic, 10 mixed<br>15 unilateral, 62 bilateral, 33 unk<br>GMFCS: 15 I, 29 II, 22 III, 29 IV, 15 V<br>MACS: 12 I, 35 II, 19 III, 28 IV, 16 V | **d Activities and Participation**<br>d5 Self-care<br>d9 Community, social and civic life; d920 Recreation and leisure<br>*Participation and Environment Measure for Children and Youth (PEM-CY):* home section | **General intellectual functioning**<br>*Revised Scale for Measuring Intelligence according to Wechsler principles (REVISK)* | **Correlation**<br>REVISK x PEMC-CY, frequency<br>Computer and video games: r = -0.294**<br>Indoor play and games: r = -0.227*<br>Arts, crafts, music and hobbies: r = -0.221*<br>Watching TV, videos and DVDs: r = -0.013<br>Getting together with people: r = -0.029<br>Socialising using technology: r = 0.035<br>Household chores: r = 0.117<br>Personal care management: r = -0.046<br>School preparation (not homework): r = -0.039<br>Homework: r = 0.308**<br>REVISK x PEMC-CY, involvement<br>Computer and video games: r = -0.226*<br>Indoor play and games: r = -0.283**<br>Arts, crafts, music and hobbies: r = -0.420**<br>Watching TV, videos and DVDs: r = -0.365**<br>Getting together with people: r = -0.331**<br>Socialising using technology: r = -0.105<br>Household chores: r = -0.075<br>Personal care management: r = -0.282**<br>School preparation (not homework): r = -0.236* |

| | | | | |
|---|---|---|---|---|
| | | | | Homework: r = -0.363** |
| Pennington et al. (2020) [35] | 77<br>2:0-2:11 years<br>25 females<br>55 spastic, 22 dyskinetic<br>16 unilateral, 39 bilateral, 22 unk<br>GMFCS: 24 I, 12 II, 6 III, 10 IV, 25 V<br>MACS: 8 I, 28 II, 13 III, 17 IV, 11 V | **d Activities and Participation**<br>d3 Communication; d 310-d329 Communicating – receiving, d330-d349 Communicating – producing, d350-d369 Conversation and use of communication devices and techniques<br>*Communication Function Classification System (CFCS)*<br>*Functional Communication Classification Scale (FCCS)* | **General intellectual functioning**<br>*Mullen Scales of Early Learning (MSEL):* visual reception scale<br>**Language**<br>*Preschool Language Scales-4th edition (PLS-4):* expressive and receptive subtests<br>*MacArthur Communicative Development Inventory (MCDI)* | **Univariable ordinal logistic regression (at 5 years)**<br>MSEL x CFCS: OR = 0.78; 95%CI = 0.72-0.84<br>MSEL x FCCS: OR = 0.79; 95%CI = 0.74-0.85<br>PLS-4 (receptive) x CFCS: OR = 0.90; 95%CI = 0.85-0.95<br>PLS-4 (receptive) x FCCS: OR = 0.89; 95%CI = 0.85-0.94<br>PLS-4 (expressive) x CFCS: OR = 0.89; 95%CI = 0.85-0.93<br>PLS (expressive) x FCCS: OR = 0.89; 95%CI = 0.84-0.93<br>MCDI words x CFCS: OR = 0.99; 95%CI = 0.98-0.99<br>MCDI words x FCCS: OR = 0.99; 95%CI = 0.99-0.99 |
| Bartonek et al. (2021) [45] | 40<br>5:0-17:0 years<br>16 females<br>37 spastic, 3 dyskinetic<br>4 unilateral, 33 bilateral, 3 unk<br>GMFCS: 17 I, 12 II, 11 III-IV | **d Activities and Participation**<br>d4 Mobility; d410 Changing basic body position, d450 Walking, d465 Moving around using equipment<br>*Gross Motor Function Classification System (GMFCS)* | **Executive functions**<br>*Corsi Block-tapping test (CBT)*<br>*Walking Corsi Test (WalCT)*<br>**Visual perception**<br>*Raven's Colored Progressive Matrices (RCPM)* | **Post-hoc analyses test (Fisher's Least Significant Difference)**<br>WalCT x GMFCS:<br>I - II: n.s.; I - III/IV: n.s.; II - III/IV: n.s.<br>CBT x GMFCS<br>I - II: n.s.; I - III/IV:*; II - III/IV: n.s.<br>RCPM x GMFCS:<br>I - II: n.s.; I - III/IV: n.s.; II - III/IV: n.s. |
| Burgess et al. (2021) [43] | 74<br>8:0-12:0 years<br>26 females<br>67 spastic, 4 dyskinetic, 3 ataxic<br>30 unilateral, 37 bilateral, 7 unk<br>GMFCS: 44 I, 18 II, 8 III, 4 IV<br>MACS: 30 I, 28 II, 16 III | **d Activities and Participation**<br>d5 Self-care<br>*Pediatric Evaluation of Disability Inventory–Computer Adaptive Test, speedy version (PEDI-CAT):* daily activities<br>d4 Mobility; d440 Fine hand use<br>*Both Hands Assessment (BoHA)*<br>*Kids Assisting Hand Assessment (AHA)* | **General intellectual functioning**<br>*Raven's Coloured Progressive Matrices (RCPM)* | **Multiple linear regression**<br>*Bilateral:*<br>PEDI-CAT x BoHA + RCPM + Age + BRIEF-BRI + Conners 3:<br>adjusted $r^2 = 0.68$<br>RCPM: estimate = 0.11; 95% CI =0.05-0.16**<br>*Unilateral:*<br>PEDI-CAT x AHA + RCPM + Age + BRIEF-BRI + Conners 3:<br>adjusted $r^2 = 0.40$<br>RCPM: estimate = 0.00; 95% CI =-0.09-0.09<br>*Total:*<br>PEDI-CAT x AHA + BoHA + BCP (v UCP) + RCPM + RCPM # BCP (v UCP) + Age + BRIEF-BRI + Conners 3, innattention:<br>adjusted $r^2 = 0.68$<br>RCPM: estimate: 0.11; 95% CI = 0.06-0.16** |

| | | | | |
|---|---|---|---|---|
| Koopmans et al. (2022) [32] | 35<br>9:8-18:1 years<br>16 females<br>20 spastic, 4 ataxic, 3 mixed, 1 other, 7 unk<br>6 unilateral, 14 bilateral, 15 unk<br>GMFCS: 9 I, 10 II, 6 III, 4 IV, 6 V<br>MACS: 7 I, 15 II, 5 III, 4 IV, 4 V | **d Activities and Participation**<br>d3 Communication<br>d310 - d329 Communicating – receiving, d330-d349 Communicating – producing, d350-d369 Conversation and use of communication devices and techniques<br>*Communication Function Classification System (CFCS)* | **General intellectual functioning**<br>*Leiter International Performance Scale-Revised (Leiter-R)*<br>**Language**<br>*Test for Reception of Grammar-2nd edition (TROG-2)*<br>*Test for Auditory Comprehension of Language-4th edition (TACL-4)*<br>*Peabody Picture Vocabulary Test-4th edition (PPVT-4)* | **Chi-square**<br>Leiter-R x CFCS: $\chi^2(2) = 6.350$*<br>TROG-2, TACL-4, PPVT-4 x CFCS: n.s.**Mann-Whitney U**<br>Leiter-R x CFCS I vs. II: U = 21.5**<br>Leiter-R x CFCS I vs. III: U = 2* |
| Asano et al. (2023) [33] | 45<br>4:0 -18:0 years<br>15 females<br>43 spastic, 1 dyskinetic, 1 ataxic<br>6 unilateral, 37 bilateral, 2 unk<br>GMFCS: 13 I, 12 II, 13 III, 7 IV<br>MACS: 15 I, 23 II, 6 III, 1 IV | **d Activities and Participation**<br>d4 Mobility; d410-d429 Changing and maintaining body position; d450-d469 Walking and moving<br>*Gross Motor Function Classification System-Expanded and Revised (GMFCS-E&R)*<br>d440 Fine hand use<br>*Manual Ability Classification System (MACS)*<br>d3 Communication; d310 - d329 Communicating – receiving, d330-d349 Communicating – producing, d350-d369 Conversation and use of communication devices and techniques<br>*Communication Function Classification System (CFCS)* | **General intelligence functioning**<br>*Raven's Coloured Progressive Matrices (RCPM)*<br>**Language**<br>*Picture Vocabulary Test (PVT)* | **Partial correlation analysis controlling for chronological age**<br>RCPM x GMFCS: r = -0.381*<br>RCPM x MACS: r = -0.445**<br>RCPM x CFCS: r = -0.385**<br>PVT x GMFCS: r = 0.025<br>PVT x MACS: r = -0.111<br>PVT x CFCS: r = -0.474** |
| Pierce et al. (2023) [55] | 41<br>1:1-2:11 years<br>18 females<br>CP type unk<br>CP pattern unk<br>GMFCS: 4 I, 19 II, 9 III, 9 IV | **e Environmental Factors**<br>e4 Attitudes; e410 Individual attitudes of immediate family members<br>*Family Empowerment Scale (FES):* empowerment in family, empowerment in service systems, and empowerment in community/political environments | **Cognitive development**<br>*Bayley Scales of Infant and Toddler Development-3rd edition (BSID-III):* cognitive scale | **Correlation**<br>BSID-III x FES<br>Total: r = 0.35*<br>Family scale: r = 0.19<br>Service systems scale: r = 0.09<br>Community scale: r = 0.47** |

| | | | | |
|---|---|---|---|---|
| Fontes et al. (2025) [39] | 190<br>2:0-14:0 years<br>82 females<br>CP type unk<br>50 unilateral, 140 bilateral<br>GMFCS: 33 I, 32 II, 24 III, 26 IV, 75 V | **d Activities and Participation**<br>d4 Mobility; d410-d429 Changing and maintaining body position, d450-d469 Walking and moving<br>*Gross Motor Function Classification System Family Report Questionnaire (GMFCS-FR)* | **Cognitive development**<br>*Pediatric Evaluation of Disability Inventory – Computer Adaptive Test, speedy version (PEDI-CAT):* social/cognitive skills | **Chi-square**<br>PEDI-CAT (social/cognitive skills within/lower-than-expected) x GMFCS-RF (able/not able to walk): $\chi^2 = 2.86$ |

Abbreviations: **, p ≤ 0.01; *, p ≤ 0.05; 95% CI, 95% confidence interval; AAC, Augmentative and Alternative Communication; BCP, bilateral cerebral palsy; BRIEF-BRI, Behavior Rating Inventory of Executive Functions-Behaviour Regulation Index; CI, confidence interval; CP, cerebral palsy; FI, full inclusion; GMFCS, Gross Motor Function Classification System; GMFM, Gross Motor Function Measure; ICF, International Classification of Functioning, Disability and Health; IQ, intelligence quotient; JTTHF, Jebsen–Taylor Test of Hand Function; MACS, Manual Ability Classification System; n.a., not available; n.s., not significative; OR, odds ratio; SC, self-contained classes; SE, standard error; UCP, unilateral cerebral palsy; unk, unknown.

Note: Only the statistics used in the context of this review are shown.
