# Supplementary material for: The Role of Cognitive Functioning in the ICF Framework: A Systematic Review of Its Influence on Activities and Participation and Environmental Factors in People with Cerebral Palsy
Source: J Clin Med. 2025 Sep 10;14(18):6393. doi: 10.3390/jcm14186393 (PMC12470702; doi:10.3390/jcm14186393)
Supplement: Supplementary file 1 [file jcm-14-06393-s001.zip › Supplementary Table S4.pdf]

**Supplementary Table S4.** Detailed information of articles exploring the effects of health interventions on cognition.

| Reference | Participants | ICF | Intervention | Cognitive Assessment | Outcomes |
|---|---|---|---|---|---|
| Authors (year)<br>[reference] | n (IG/CG)<br>Age range (years:months)<br>n females (IG/CG)<br>n type CP<br>n pattern CP<br>Motor ability | **ICF component**<br>ICF chapter; ICF second level | *Intervention's name*<br>Description of intervention | **Cognitive domain**<br>*Instrument*: subscales | **Statistical method**<br><u>Cognitive assessment</u><br>Results |
| Hardy et al. (2002) [64] | 75 (40/35)<br>4:0-12:0 years<br>41 females (21/20)<br>35 spastic<br>1 unilateral, 38 bilateral, 1 unk<br>Motor ability unk | **e Environmental Factors**<br>e5 Services, systems and policies; e580 Health services, systems and policies | *Hyperbaric oxygen treatment ($HBO_2$)*<br>IG: 100% oxygen at 1.75 atmospheres absolute ($HBO_2$)<br>CG: air (21% oxygen) at 1.3 atmospheres absolute (Sham)<br>40 sessions of 1 hour of either $HBO_2$ or Sham treatment, for 2 months | **Executive functions**<br>*Corsi Blocks (CB)*<br>*Picture Span Tests*<br>*Word Span Test*<br>*Test of Variables of Attention (TOVA), 10.8-minute vigilant condition:* correct non-responses<br>**Attention**<br>*Test of Variables of Attention (TOVA), 10.8-minute vigilant condition:* correct responses, reaction time (ms), variability in reaction (ms) | **t-test**<br>*Pre-treatment x post-treatment:*<br><u>CB:</u><br>$HBO_2$,: mean difference = +0.61*<br><u>Picture Span:</u><br>$HBO_2$,: mean difference = +0.7**<br><u>Word Span (familiar words):</u><br>$HBO_2$,: mean difference = -0.19<br><u>Word Span (unfamiliar words):</u><br>$HBO_2$,: mean difference = -0.04<br><u>TOVA (auditory correct responses):</u><br>$HBO_2$,: mean difference = +7.5*<br><u>TOVA (auditory reaction time, ms):</u><br>$HBO_2$,: mean difference = +57<br><u>TOVA (auditory variability in reaction time, ms):</u><br>$HBO_2$,: mean difference = +11<br><u>TOVA (auditory correct non-responses):</u><br>$HBO_2$,: mean difference = +44.8**<br><u>TOVA (visual correct responses):</u><br>$HBO_2$,: mean difference = +3<br><u>TOVA (visual reaction time, ms):</u><br>$HBO_2$,: mean difference = +22<br><u>TOVA (visual variability in reaction time, ms):</u><br>$HBO_2$,: mean difference = -32<br><u>TOVA (visual correct non-responses):</u><br>$HBO_2$,: mean difference = +41**<br>*Pre-treatment x follow-up (3m):*<br><u>CB:</u><br>$HBO_2$,: mean difference = +1.1**<br><u>Picture Span:</u><br>$HBO_2$,: mean difference = +0.56*<br><u>Word Span (familiar words):</u><br>$HBO_2$,: mean difference = +0.73** |

| | | | | | |
|---|---|---|---|---|---|
| | | | | | <u>Word Span (unfamiliar words):</u><br>$HBO_2$,: mean difference = +0.15<br><u>TOVA (auditory correct responses):</u><br>$HBO_2$,: mean difference = +10.1**<br><u>TOVA (auditory reaction time, ms):</u><br>$HBO_2$,: mean difference = +55<br><u>TOVA (auditory variability in reaction time, ms):</u><br>$HBO_2$,: mean difference = -19<br><u>TOVA (auditory correct non-responses):</u><br>$HBO_2$,: mean difference = +42.9<br><u>TOVA (visual correct responses):</u><br>$HBO_2$,: mean difference = +5.2<br><u>TOVA (visual reaction time, ms):</u><br>$HBO_2$,: mean difference = +116<br><u>TOVA (visual variability in reaction time, ms):</u><br>$HBO_2$,: mean difference = -7<br><u>TOVA (visual correct non-responses):</u><br>$HBO_2$,: mean difference = +53.2** |
| Maltais et al. (2016) [60] | 17 (8/9)<br>6:3-12:5 years<br>7 females (3/4)<br>8 spastic<br>CP pattern unk<br>GMFCS: 8 I | **e Environmental Factors**<br>e5 Services, systems and policies; e580 Health services, systems and policies | *Intense exercise*<br>Intense aerobic exercise that consists of a shuttle run and walk test<br>1 session | **Executive functions/Processing speed**<br>*Stroop-like test, modified designed for children* | **Paired t-test**<br>*Pre-exercice x post-exercice*:<br><u>Response accuracy x intensive exercise:</u><br>pre: mean ± SD = 96.3 ± 1.09%, post: mean ± SD = 95.1 ±0.96%; t (1,7) = 1.158<br><u>Reaction time (RT) x intensive exercise:</u><br>pre: mean ± SD = 892 ± 56.5ms, post: mean ± SD = 798 ± 45.6ms; t (1,7) = 4.411**<br><u>Interference effect x intensive exercise:</u><br>pre: mean ± SD = 4.5 ± 2.5%RT, post: mean ± SD = 13 ± 2.9%RT; t (1,7) = -2.167* |
| Teixeira-Machado et al. (2017) [24] | 26 (13/13)<br>15:0-29:0 years<br>15 females (8/7)<br>CP type unk<br>CP pattern unk<br>GMFCS: 3 II, 5 III, 4 IV, 1 V | **e Environmental Factors**<br>e5 Services, systems and policies; e580 Health services, systems and policies | *Dance intervention*<br>Physical intervention (coordination-movements of upper and lower limbs, body image interaction between subject and environment, skill and agility sequential components of the movement and trunk and head movements for spatial orientation and equilibrium)<br>2 sessions of 60 minutes, twice per week, for 3 months | **Cognitive development**<br>*Functional Independence Measure (FIM):* cognitive function | **t-test for dependent and independent samples**<br><u>FIM:</u> pre: mean ± SD = 2.71 ± 0.46, post: mean ± SD = 5.38 ± 0.50* |

| | | | | | |
|---|---|---|---|---|---|
| Mak et al. (2018) [58] | 42 (21/21)<br>6:0-16:0 years<br>18 females (7/11)<br>21 spastic<br>7 unilateral, 14 bilateral<br>GMFCS: 11 I, 4 II, 6 III | **e Environmental Factors**<br>e5 Services, systems and policies; e580 Health services, systems and policies | *MiYoga*<br>Mindfulness and mindful movement techniques based on hatha yoga principles<br>6 sessions of 90 minutes, for 6 weeks with plus 2 follow-up consultations via phone or Skype over the following 2 weeks | **Attention**<br>*Conners' Continuous Performance Test-2nd edition (CPT-II)*<br>*Wechsler Intelligence Scale for Children-4th edition (WISC-IV):* symbol search<br>**Executive functions**<br>*Wechsler Intelligence Scale for Children-4th edition (WISC-IV):* digit span (DS)<br>*Delis-Kaplan Executive Function System (D-KEFS):* colour word interference (CWIT), trail making test (TMT) | **Covariance**<br>*Postintervention MiYoga x CG:*<br><u>CPT-II</u><br>Hit reaction time block change: F = 3.74; $\eta_p^2$ = 0.10; treatment effect = -5.82; 95%CI = -11.95- 0.31<br>Hit reaction time: F = 0.43; $\eta_p^2$ = 0.01; treatment effect = -1.71; 95%CI = -6.99-3.58<br>Hit reaction time standard error: F = 4.59*; $\eta_p^2$ = 0.13; treatment effect = -5.24; 95%CI = -10.22--0.26<br>Commissions: F = 1.34; $\eta_p^2$ = 0.04; treatment effect = -3.01; 95%CI = -8.30-2.28<br>Omissions: F = 1.55; $\eta_p^2$ = 0.05; treatment effect = -8.00; 95%CI = -21.10-5.11<br>Detectability reaction time for target vs non-target: F= 0.03; $\eta_p^2$ = < 0.01; treatment effect = -0.66; 95%CI = -8.26-6.95<br>Variability of reaction time: F = 1.80; $\eta_p^2$ = 0.05; treatment effect = -3.10; 95%CI = -7.83-1.62<br>Hit reaction time interstimulus interval change: F = 0.42; $\eta_p^2$ = 0.01; treatment effect = -2.14; 95%CI = -8.87-4.58<br>Perseverations: F = 4.60*; $\eta_p^2$ = 0.13; treatment effect = -9.49; 95%CI = -18.50--0.48<br>Response style: F = 0.02; $\eta_p^2$ = < 0.01; treatment effect = 0.57; 95%CI = -7.75-8.89<br><u>WISC-IV</u><br>Symbol search: F = 0.24; $\eta_p^2$ = < 0.01; treatment effect = -0.41; 95%CI = -2.09-1.28<br>DS total: F = 0.19; $\eta_p^2$ = < 0.01; treatment effect = -0.39; 95%CI = -2.20-1.42<br><u>D-KEFS</u><br>CWIT (condition 3; total errors): F = 1.23; $\eta_p^2$ = 0.07; treatment effect = -2.00; 95%CI= -5.82-1.81<br>CWIT (condition 3; time): F = 2.32; $\eta_p^2$ = 0.12; treatment effect = -1.91; 95%CI = -4.54-0.73<br>CWIT (condition 4; total errors): F = 0.03; $\eta_p^2$ = <0.01; treatment effect = -0.33; 95%CI= -4.31-3.65<br>CWIT (condition 4; time): F = 0.26; $\eta_p^2$ = 0.02; treatment effect = -0.32; 95%CI = -1.63-1.00<br>TMT (condition 4; total errors): F = 0.28; $\eta_p^2$ = 0.02; treatment effect = -0.45; 95%CI = -1.36-2.26<br>TMT (condition 4; time): F = 0.08; $\eta_p^2$ = <0.01; treatment effect = 0.47; 95%CI = -3.11-4.05 |

| | | | | | |
|---|---|---|---|---|---|
| Alwhaibi et al. (2020) [61] | 45 (15/30)<br>5:5-7:9 years<br>22 females (5/CGa: 10, CGb: 7)<br>15 spastic<br>15 unilateral<br>MACS: 15 I-II | **e Environmental Factors**<br>e5 Services, systems and policies; e580 Health services, systems and policies | *Augmented biofeedback*<br>E-Link Upper Limb Exerciser, a computerized graded interactive system<br>*Physical training*<br>Exercises facilitating hand–eye coordination and fine motor skills<br>1 session of 60 minutes per day, three times per week, for 3 months | **Visual perception**<br>*Beery-Buktenica Developmental Test of Visual-Motor Integration-6$^{th}$ edition (Beery):* visual-motor integration (VMI), visual perception (VP) | **Mixed MANOVA**<br>*Pre-treatment x post-treatment:*<br>Beery VMI: pre: mean ± SD = 81.13 ± 4.05, post: mean ± SD = 115.33 ± 5.24**<br>Beery VP: pre: mean ± SD = 79.86 ± 3.72, post: mean ± SD = 112.8 ± 4.41** |
| Mak et al. (2022) [59] | 23<br>6:0-16:0 years<br>0 females<br>23 spastic<br>10 unilateral, 13 bilateral<br>GMFCS: 14 I, 6 II, 3 III | **e Environmental Factors**<br>e5 Services, systems and policies; e580 Health services, systems and policies | *MiYoga*<br>Mindfulness and mindful movement techniques based on hatha yoga principles<br>6 sessions of 90 minutes, for 6 weeks with plus 2 follow-up consultations via phone or Skype over the following 2 weeks | **Attention**<br>*Conners' Continuous Performance Test-2$^{nd}$ edition (CPT-II)*<br>*Wechsler Intelligence Scale for Children-4$^{th}$ edition (WISC-IV):* symbol search<br>**Executive functions**<br>*Wechsler Intelligence Scale for Children-4$^{th}$ edition (WISC-IV):* digit span (DS)<br>*Delis-Kaplan Executive Function System (D-KEFS):* color word interference (CWIT), trail making test (TMT) | **Paired t-test**<br>*Pre-intervention x follow-up:*<br>CPT-II<br>Hit reaction time block change): t = 0.86<br>Hit reaction time: t = 1.14<br>Hit reaction time standard error: t = 0.85<br>Commissions: t = 0.17<br>Omissions: t = 1.34<br>Detectability reaction time for target vs non-target: t = 1.05<br>Variability of reaction time: t = 0.65<br>Hit reaction time interstimulus interval change: t = 0.96<br>Perseverations: t = -0.08<br>Response style: t = 0.26<br>WISC-IV<br>Symbol search: t = -0.66<br>DS total: t = -0.66<br>D-KEFS<br>CWIT (condition 3; total errors): t = -1.95<br>CWIT (condition 3; time): t = -3.17**<br>CWIT (condition 4; total errors): t = -1.42<br>CWIT (condition 4; time): t = -3.69**<br>TMT (condition 4; total errors): t = -1.33<br>TMT (condition 4; time): t = -2.16<br>*Post-intervention x follow-up:*<br>CPT-II<br>Hit reaction time block change: t = 0.06<br>Hit reaction time: t = 0.87<br>Hit reaction time SE: t = -0.62<br>Commissions: t = -1.65 |

| | | | | | |
|---|---|---|---|---|---|
| | | | | | Omissions: t = 1.48<br>Detectability reaction time for target vs non-target: t = -0.80<br>Variability of reaction time: t = -0.85<br>Hit reaction time interstimulus interval change: t = 0.23<br>Perseverations: t = -0.54<br>Response style: t = 0.32<br>WISC-IV<br>Symbol search: t = 0.51<br>DS total: t = -0.70<br>D-KEFS<br>CWIT (condition 3; total errors): t = -0.70<br>CWIT (condition 3; time): t = -2.11<br>CWIT (condition 4; total errors): t = 0.06<br>CWIT (condition 4; time): t = -1.83<br>TMT (condition 4; total errors): t = -0.59<br>TMT (condition 4; time): t = 1.19 |
| Collange-Grecco et al. (2023) [63] | 30 (15/15)<br>6:0-12:0 years<br>unk females<br>15 spastic<br>4 unilateral, 9 bilateral<br>GMFCS: 4 I, 7 II, 4 III | **e Environmental Factors**<br>e5 Services, systems and policies; e580 Health services, systems and policies | *Transcranial Direct Current Stimulation (tDCS)*<br>tDCS combined with treadmill training and training of intellectual activities<br>10 sessions | **General intelligence functioning**<br>*Raven's Coloured Progressive Matrices (RCPM)* | **Two-way ANOVA**<br>*IG vs. CG:*<br>Post-intervention: RCPM: IG: mean ± SD = 26.2 ± 2.5; CG: mean ± SD = 24.4 ± 2.3<br>Follow-up: RCPM: IG: mean ± SD = 29 ± 1.5*; CG: mean ± SD = 24.5 ± 2.4 |
| AL-Nemr (2024) [57] | 32 (16/16)<br>8:0-12:0 years<br>14 females (9/5)<br>16 spastic<br>16 bilateral<br>GMFCS: 6 II, 10 III | **e Environmental Factors**<br>e5 Services, systems and policies; e580 Health services, systems and policies | *Functional Strength Training (FST)*<br>Functional strength training for lower limbs followed by conventional physical therapy<br>3 sessions of 90 minutes each per week, for 6 months | **Attention/Processing speed/ Memory/Executive functions**<br>*Computer-based RehaCom software* | **Mixed ANOVA**<br>RehabCom<br>Attention: pre: mean ± SD = 4.48 ± 0.76; post: mean ± SD = 4.18 ± 0.63; follow-up: mean ± SD = 4.41 ± 0.70**<br>Figural memory: pre: mean ± SD = 13.03 ± 1.28; post: mean ± SD = 12.16 ± 1.14; follow-up: mean ± SD = 12.83 ± 1.11**<br>Reaction behaviour: pre: mean ± SD = 3.20 ± 1.24; post: mean ± SD = 2.84 ± 1.17; follow-up: mean ± SD = 3.06 ± 1.23**<br>Logical reasoning: pre: mean ± SD = 16.28 ± 1.38; post: mean ± SD = 15.18 ± 1.65; follow-up: mean ± SD = 15.88 ± 1.29** |
| Chen et al. (2024) [62] | 19 (8/11)<br>4:0-12:0 years<br>2 females (1/1) | **e Environmental Factors**<br>e5 Services, systems and policies; e580 Health | *EEG Neurofeedback training*<br>Neurofeedback | **Attention**<br>*Conners Continuous Performance Test-2nd edition (CPT-II)* | **ANCOVA**<br>*CG and IG in post-treatment (covariates pre-test scores and age):* |

| | | | | | |
|---|---|---|---|---|---|
| | CP type unk<br>4 unilateral, 4 bilateral<br>GMFCS: 4 I, 1 II, 3 III<br>MACS: 3 I, 5 II | services, systems and policies | 2 sessions of approximately for 1 hour each, for 10 weeks | **Visual perception**<br>*Test of Visual-Perceptual Skills-3$^{rd}$ edition (TVPS-3)* | <u>CPT</u><br>Confidence: η2 = 0.01<br>Omission: η2 = 0.13<br>Commission: η2 = 0.01<br>Hit RT: η2 = 0.04<br>Variability: η2 = 0.02<br>Detectability: η2 = 0.02<br><u>TVPS-3</u><br>Visual discrimination: η2 = 0.10<br>Visual memory: η2 = 0.04<br>Visual spatial relationship: η2 = 0.00<br>Visual form constancy: η2 = 0.00<br>Visual sequential memory: F = 5.20; η2 = 0.25*<br>Visual figure-ground: η2 = 0.01<br>Visual closure: F = 8.88; η2 = 0.36** |

Abbreviations: **, $p \leq 0.01$; *, $p \leq 0.05$; 95% CI, 95% confidence interval; ANCOVA, analysis of covariance; ANOVA, analysis of variance; CG, control group; CP; cerebral palsy; GMFCS, Gross Motor Function Classification System; ICF, International Classification of Functioning, Disability and Health; IG, intervention group; MACS, Manual Ability Classification System; MANOVA, multivariate analysis of variance; SD, standard deviation; SE, standard error; unk, unknown.

Note*:* Only the statistics used in the context of this review are shown.
