# Supplementary material for: The Role of Cognitive Functioning in the ICF Framework: A Systematic Review of Its Influence on Activities and Participation and Environmental Factors in People with Cerebral Palsy
Source: J Clin Med. 2025 Sep 10;14(18):6393. doi: 10.3390/jcm14186393 (PMC12470702; doi:10.3390/jcm14186393)
Supplement: Supplementary file 1 [file jcm-14-06393-s001.zip › Supplementary Table S5.pdf]

Supplementary Table S5. Detailed information of articles exploring the effects of cognitive interventions on non-cognitive aspects of functioning.

Reference	Participants	ICF	Intervention	ICF Assessment	Outcomes
Authors (year)<br>[reference]	n (IG/CG)<br>Age range (years:months)<br>n females (IG/CG)<br>n type CP<br>n pattern CP<br>Motor ability	**ICF component**<br>ICF chapter; ICF second level	*Intervention's name*<br>Description of intervention	**ICF component**<br>ICF chapter; ICF second level<br>*Assessment*: subscales	**Statistical method**<br><u>ICF assessment</u><br>Results
Muriel et al. (2014) [40]	15<br>7:0-14:0 years<br>7 females<br>14 spastic, 1 ataxic<br>7 unilateral, 8 bilateral<br>GMFCS: 6 I, 4 II, 2 III, 3 V	**e Environmental Factors**<br>e5 Services, systems and policies; e580 Health services, systems and policies	*Guttman NeuroPersonalTrainer, child version*<br>Online and individual intervention, adapted depending on the cognitive function baseline level<br>16 sessions of 1 hour, 2 days a week, for 8 weeks	**d Activities and Participation**<br>d2 General tasks and demands; d230 Carrying out daily routine<br>*Conners rating scales (CPRS-48 / CTRS-28)*<br>*Behavior Rating Inventory of Executive Function (BRIEF)*	**Wilcoxon signed-rank test**<br>*Pre-treatment x post-treatment:*<br><u>CPRS-48:</u> n.s.<br><u>CTRS-28</u>: n.s.<br><u>BRIEF:</u> n.s.
Beneventi et al. (2023) [65]	66 (32/34)<br>11.4y ± 3.1y/9.4y ± 2.6y<br>25 females (13/12)<br>CP type unk<br>CP pattern unk<br>Motor ability unk	**e Environmental Factors**<br>e5 Services, systems and policies; e580 Health services, systems and policies	*CogMed RM computer program*<br>Computerized cognitive training<br>Around 25 sessions of 30-40 minutes, 5 days a week, for 5 weeks	**d Activities and Participation**<br>d2 General tasks and demands; d230 Carrying out daily routine<br>*ADHD rating scale IV*<br>*Behaviour Rating Inventory of Executive Function (BRIEF)*	**ANCOVA**<br>*IG vs. CG after training:*<br><u>ADHD rating scale IV:</u> n.s.<br><u>BRIEF:</u> n.s.
Wotherspoon et al. (2024) [67]	21 (9/12)<br>4 females (whole semple)<br>8:3-12:6 years<br>CP type unk<br>CP pattern unk<br>Motor ability unk	**e Environmental Factors**<br>e5 Services, systems and policies; e580 Health services, systems and policies	*Strengthening Mental Abilities Through Relational Training (SMART)*<br>Online cognitive training program<br>Participants could complete 5 modules per day with a total of 55 modules to complete up to 12 weeks	**d Activities and Participation**<br>d2 General tasks and demands; d230 Carrying out daily routine<br>*Conners-3 Rating Scale*<br>*Behaviour Rating Inventory of Executive Function (BRIEF)*<br>d3 Communication; d350-369 Conversation and use of communication devices and techniques<br>*Social Communication Questionnaire – Current, (SCQ – Current – Parent form)*	**Paired t tests**<br><u>Conners-3</u><br>Innatention: t = 0.747<br>Hyperactivity/impulsivity: t = -1.59<br>Learning problems: t = -1.019<br>Executive function: t = 0.311<br>Peer relations: t= -1.286<br><u>BRIEF:</u> t = -1.418<br><u>SCQ:</u> t = -0.962<br><u>SDQ:</u> t = 0.480<br><u>BASC-3</u><br>Externalising problems: t= -0.810<br>Internalising problems: t= -0.534<br>Behavioural symptom index: t= -0.611<br>Adaptive skills: t = 0.589

				d7 Interpersonal interactions and relationships; d720 Complex interpersonal interactions<br>*Strengths and Difficulties Questionnaire (SDQ)*<br>*Behavior Assessment System for Children–3rd edition (BASC-3)*	
Blasco et al. (2025) [66]	60 (30/30)<br>8:11-12:11 years<br>30 females (15/15)<br>27 spastic, 3 dyskinetic<br>17 unilateral, 10 bilateral, 3 unk<br>GMFCS: 20 I, 6 II, 4 III<br>MACS: 11 I, 16 II, 3 III	**e Environmental Factors**<br>e5 Services, systems and policies; e580 Health services, systems and policies	*Neuronup*<br>Home-based computerized executive function intervention<br>10 sessions of 15 minute per week, for 12 weeks	**d Activities and Participation**<br>d9 Community, social and civic life; d920 Recreation and leisure<br>*Participation and Environment Measure for Children and Youth Questionnaire (PEM-CY):* home, school and community	**ANCOVA**<br>*Post intervention:*<br><u>PEMCY (home), PSS and FQOL covariate:</u> estimated marginal mean ± SD = 5.76 ± 0.12; mean difference = 0.17; 95%CI = -0,19-0,52; F = 0,9; η_p^2 = 0.02<br><u>PEMCY (school):</u> estimated marginal mean ± SD = 4.00 ± 0.24; mean difference = -0.18; 95%CI = -0.87-0.50; F = 0.28; η_p^2 = 0.01<br><u>PEMCY (community):</u> estimated marginal mean ± SD = 2.96 ± 0.18; mean difference = 0.20; 95%CI = −0.31-0.72; F = 0.29; η_p^2 = 0.01<br>*Follow-up (9 months after intervention):*<br><u>PEMCY (home), PSS and FQOL covariate:</u> estimated marginal mean ± SD = 5.57 ± 0.14; mean difference = 0.05; 95%CI = -0.33-0.42; F = <0.01; η_p^2 <0.01<br><u>PEMCY (school):</u> estimated marginal mean ± SD = 4.20 ± 0.20; mean difference = 0.10; 95%CI = −0.4-0.67; F = 0.12; η_p^2 <0.01<br><u>PEMCY (community):</u> estimated marginal mean ± SD = 2.48 ± 0.15; mean difference = -0.17; 95%CI = -0.60-0.26; F = 0.66; η_p^2 = 0.01

Abbreviations: **, p ≤ 0.01; *, p ≤ 0.05; 95% CI, 95% confidence interval; ADHD, Attention-Deficit/Hyperactivity Disorder; ANCOVA, analysis of covariance; CG, control group; CP; cerebral palsy; CPRS-48, Conners Parent Rating Scale – 48 items; CTRS-28, Conners Teacher Rating Scale – 28 items; FQOL, Family Quality of Life Scale; GMFCS, Gross Motor Function Classification System; ICF, International Classification of Functioning, Disability and Health; IG, intervention group; MACS, Manual Ability Classification System; n.s., not significative; PSS, Parental Stress Scale; SD, standard deviation; unk, unknown.

Note*:* Only the statistics used in the context of this review are shown.
